# Enhancing patient knowledge and behaviour through digital health communication: a systematic review and random-effects meta-analysis of mobile, web-based, social media, telehealth, and AI-enabled interventions

**DOI:** 10.3389/fpubh.2026.1741936

**Published:** 2026-06-24

**Authors:** Asma Alshanqiti, Hadel Alghabban, Amal Mohammed Q. Surrati, Sara Ibrahim, Wejdan Owaydhah

**Affiliations:** 1 Department of Family and Community Medicine and Medical Education, Taibah University College of Medicine, Medina, Saudi Arabia; 2 Department of Basic Medical Sciences, Taibah University College of Medicine, Medina, Saudi Arabia

**Keywords:** artificial intelligence, digital health communication, digital health literacy, meta-analysis, mobile apps, patient empowerment, social media, systematic review

## Abstract

**Background:**

Digital technologies are increasingly used to support health communication, yet their impact on knowledge, behaviour, and health literacy outcomes remains unclear. Previous reviews have often conflated improvements in knowledge with digital health literacy, without carefully distinguishing skills from outcomes.

**Objective:**

To systematically review and synthesize evidence on the effectiveness of digital health communication interventions—including mobile apps, web platforms, social media, telehealth/video tools, and emerging AI-enabled approaches—on knowledge, behaviour, clinical, and empowerment outcomes, while distinguishing these outcomes from digital health literacy when directly measured.

**Methods:**

We searched PubMed, Embase, Scopus, CINAHL, Web of Science, and Cochrane Library (January 2015 to January 2026). Eligible studies included randomized and non-randomized trials evaluating digital interventions designed to improve health knowledge, literacy, behaviours, or related outcomes. Only English-language, peer-reviewed studies were included; grey literature, conference abstracts, dissertations, preprints, and unpublished studies were not systematically searched. Random-effects meta-analysis was performed where outcomes were comparable, with subgroup and sensitivity analyses. For subgroup analyses, overlapping multi-component interventions were assigned once using a prespecified dominant patient-facing modality rule based on the main user interface and delivery channel; AI-enabled status was applied only when AI materially informed personalization or decision support. Certainty of evidence was graded using GRADE.

**Results:**

Thirty studies met inclusion criteria, and 12 contributed to at least one pooled meta-analysis. Continuous knowledge/literacy outcomes showed a small, statistically non-significant pooled effect (SMD 0.25; 95% CI: −0.04 to 0.54) with substantial heterogeneity. Dichotomous behavioural outcomes showed a statistically significant improvement (OR 3.57; 95% CI: 1.60 to 7.97), but this estimate was based on only two studies and should be interpreted cautiously. Mobile and web-based interventions provided the most frequent directionally favourable evidence, whereas AI-enabled, social media, and telehealth/video modalities were sparsely represented. Clinical and empowerment outcomes were too heterogeneous for pooling and showed mixed findings. GRADE certainty was low for knowledge/literacy, clinical, and empowerment outcomes and moderate only for selected behavioural outcomes.

**Conclusion:**

Digital health communication interventions may support patient knowledge and selected health behaviours; however, the strength of evidence remains limited by high heterogeneity, small numbers of pooled studies, overlapping intervention features, risk of bias, variable outcome measures, and exclusion of grey literature and non-English studies. Stronger conclusions about effectiveness, particularly for AI-enabled, social media, and telehealth/video approaches, require adequately powered trials using standardized literacy, behavioural, clinical, and empowerment outcomes.

**Systematic review registration:**

PROSPERO registration number: CRD42024625595.

## 1 Introduction

The transformation in the creation, dissemination, and use of health information is driven by digital technologies, including wearable devices, social media, telehealth platforms, mobile applications, and conversational agents (1, 2). This enables personalized support for behaviour change, interactive education, and targeted messaging (3, 4). More recently, advances in artificial intelligence (AI), such as machine learning, natural language processing, and adaptive decision-support systems, have expanded the potential for digital technologies to deliver scalable, customized, and timely interventions (5). With issues of accessibility, usability, and governance being adequately addressed, digital health is increasingly recognized by global health organizations as a critical enabler of equitable and effective health systems (6–8).

There are three related but distinct concepts: health literacy, digital health literacy, and health communication (1, 9, 10). Health literacy is a set of cognitive and social competencies that enable people to access, understand, appraise, and apply health information to make informed decisions (10, 11). Digital health literacy (DHL) extends these competencies to digital environments, including searching for, evaluating, and applying online health information or interacting with digital platforms (12, 13). Health communication is distinguished from those concepts in that the central aim is to describe and deliver strategies for informing and influencing health decisions (14, 15).

AI in health communication includes technologies designed to tailor health information, provide automated counselling via chatbots or virtual assistants, and use predictive analytics to support engagement and self-management (16, 17). While recent years have seen rapid expansion in the integration of AI, early systems, such as MYCIN developed in the 1970s, indicate this has been a long-term development rather than a recent phenomenon (18). Most digital health interventions report at least one of the following: increased screening uptake, improved treatment adherence, physical activity, or psychological well-being (19–21). Many studies also report increase in knowledge or patient empowerment (22). However, few studies have directly measured DHL with a validated tool, which precludes conclusions on whether any improvement in health outcomes has been mediated by improved literacy skills, or by other mechanisms, including better tailoring of messages, ease of access, etc. (23–25). This gap complicates interpretation but also underscores the importance of framing current evidence as an evaluation of digital communication tools and their effects on knowledge, behaviour, and health outcomes, with implications for digital health literacy where measured.

The aim of this systematic review and meta-analysis is therefore to evaluate the effectiveness of digital health communication interventions—including mobile, web-based, social media, telehealth/video, and AI-enabled tools—on knowledge, behaviour, clinical, and empowerment outcomes, while distinguishing these effects from digital health literacy unless DHL was directly measured. Specifically, the review (i) synthesizes evidence on the effects of digital interventions on knowledge, behaviour change, and health outcomes; (ii) quantifies pooled effects where comparable outcomes are available; and (iii) identifies gaps in measurement, equity, and implementation to guide future research and policy.

Operationally, this review treated the digital platform or communication channel as the intervention mechanism and treated knowledge, behaviour, clinical outcomes, empowerment, and directly measured DHL as separate outcome domains. Improvements in disease-specific knowledge, adherence, or engagement were not interpreted as evidence of improved digital health literacy unless a validated DHL instrument was used. This distinction guided eligibility decisions, outcome grouping, subgroup interpretation, and the cautious interpretation of pooled estimates.

## 2 Methods

### 2.1 Protocol and reporting

This systematic review and meta-analysis followed the PRISMA 2020 guidelines to ensure rigor and transparency (26). The study protocol was prospectively registered with PROSPERO (CRD42024625595). Any deviations from the protocol (e.g., outcome grouping, analytical refinements) are reported in the Supplementary Materials.

### 2.2 Conceptual scope and definitions

Because prior literature often conflates improvements in knowledge or behaviour with improvements in (digital) health literacy, we clarified definitions at the outset:

- Health literacy: the cognitive and social skills that enable individuals to access, understand, appraise, and apply health information in decision-making.
- Digital health literacy (DHL): the application of these competencies in digital environments (e.g., evaluating online information, using apps, or interacting with digital platforms).
- Health communication (focus of this review): digital communication strategies—including apps, web portals, telehealth, social media, and AI-enabled tools—designed to inform, motivate, or support health-related decisions.

We therefore evaluate outcomes of digital health communication interventions, and when DHL was directly measured using validated instruments (e.g., eHEALS, DHLI), it was treated as a distinct outcome and potential mediator rather than assumed proxy.

### 2.3 Eligibility criteria (PICOS)

- Population: Children, adolescents, and adults from any setting. Studies focusing on underserved groups (e.g., low-SES, rural, older adults) were eligible.
- Interventions: Digitally mediated, patient-facing health communication (apps, web-based tools, telehealth, chatbots, AI-based decision support, or social media campaigns). Multi-component programs were included if the digital communication element was central or separately evaluated.
- Comparators: Standard care, non-digital interventions, waitlist/attention control, alternative digital interventions, or pre-post without a comparator if otherwise eligible.
- Outcomes: At least one primary outcome domain—knowledge, behaviour, clinical/health outcomes, or empowerment. Studies measuring only satisfaction/usability without health outcomes were excluded. Secondary outcomes included engagement, adoption, satisfaction, and patient–provider communication.
- Study designs: Only interventional studies evaluating patient-facing digital health communication tools with measurable effects on knowledge, behaviour, clinical, or empowerment outcomes were eligible for inclusion. Validation studies, trial protocols, and secondary analyses without assessment of intervention effects were excluded from quantitative synthesis.

Report characteristics: Peer-reviewed studies published in English between January 2015 and January 2026. Conference abstracts, dissertations, preprints, and other unpublished or grey-literature sources were not included.

### 2.4 Outcome definition

Outcomes were defined *a priori* and analysed according to a hierarchical framework reflecting increasing proximity to clinical impact. The hierarchy was applied consistently across study selection, data extraction, narrative synthesis, and meta-analysis:

1. Digital Health Literacy (primary outcome): Measured exclusively using validated instruments assessing competencies related to accessing, understanding, appraising, and applying digital health information (e.g., eHEALS, DHLI, HLQ digital domains). Proxy measures and unvalidated scales were not classified as digital health literacy outcomes.
2. Knowledge outcomes: Disease-specific or health-related knowledge assessed through validated questionnaires, standardized tests, or clearly defined structured assessments. Knowledge outcomes were analysed separately from digital health literacy and were not treated as equivalent constructs.
3. Behavioural outcomes: Health-related behaviours were categorized as either objective (e.g., registry-verified screening uptake, device-recorded physical activity) or self-reported (e.g., questionnaires, activity logs). Where both were available, objective measures were prioritised.
4. Clinical outcomes: Patient-level health outcomes, including clinical indicators (e.g., biometrics, symptom scores), healthcare utilisation, or quality-of-life measures assessed using validated instruments. These outcomes were synthesised narratively due to limited homogeneity.

This hierarchy was used to prevent conceptual overlap, ensure analytical consistency, and avoid conflation of literacy, knowledge, behavioural, and clinical effects.

### 2.5 Information sources and search strategy

We searched PubMed/MEDLINE, Embase, Scopus, Web of Science, CINAHL, and Cochrane Library (January 2015–January 2026). Search terms combined subject headings and keywords for “digital health,” “health communication,” “artificial intelligence,” “behaviour change,” and “health outcomes.” Full strategies are available in Supplementary Table S1. Reference lists of included articles and relevant reviews were hand-searched to identify additional studies. Grey literature sources, conference abstracts, dissertations, preprints, and unpublished studies were not systematically searched.

### 2.6 Study selection

**Figure 1:**
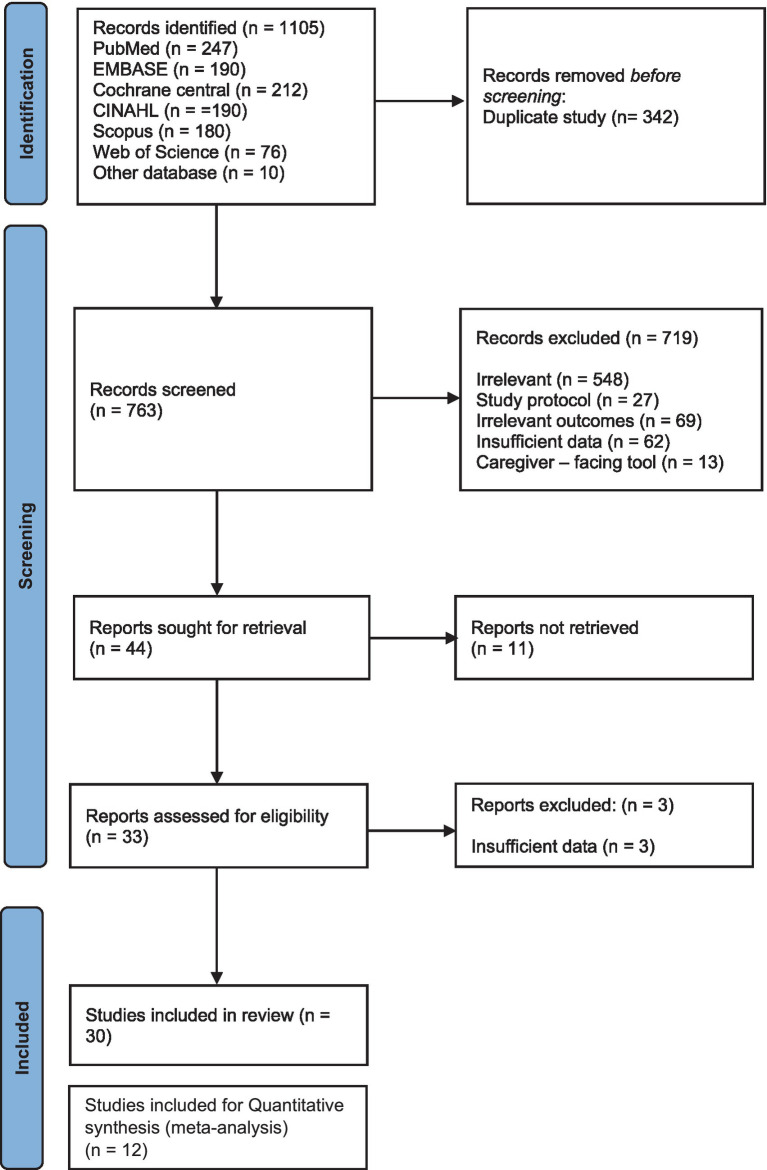
Study flow diagram using the preferred reporting items for systematic reviews and meta-analyses.

Two reviewers independently screened titles/abstracts and then full texts against eligibility criteria. Disagreements were resolved by discussion or a third reviewer. Cohen’s *κ* was calculated at both stages. Full-text exclusion reasons were recorded and mapped to PRISMA categories (e.g., no eligible outcomes, insufficient data, no patient-facing communication, wrong comparator, non-interventional design, non-English). The PRISMA 2020 flow diagram (Figure 1) summarizes identification, screening, eligibility, and inclusion.

### 2.7 Data extraction

Using a piloted template, two reviewers independently extracted relevant data from each included study. This process encompassed study metadata such as publication year, country, study design, setting, and sample size. Participant characteristics were also recorded, including age, sex, socioeconomic status (SES), and health status. Intervention details were documented thoroughly, covering the type of technology used, presence of AI features, duration of the intervention, mode of delivery, and any theoretical framework underpinning the approach. Information on comparators was noted, along with outcomes measured using validated scales where applicable, the tools used for measurement, and the timing of follow-up assessments. Effect sizes or data suitable for calculating them were extracted, and funding sources or conflicts of interest were recorded. In cases where multiple reports described the same study, data were consolidated to avoid duplication. When information was incomplete, study authors were contacted twice for clarification. Any disagreements between reviewers were resolved through consensus.

### 2.8 Risk of bias and quality assessment

Risk of bias was assessed using appropriate tools based on study design. For randomized controlled trials (RCTs) and cluster RCTs, the Cochrane Risk of Bias 2 (RoB 2) tool was applied (27). Non-randomized studies were evaluated using the ROBINS-I tool, while pre–post studies without a control group were assessed using either the NIH Before–After Tool or the JBI checklist (28, 29). Two reviewers conducted the assessments independently, with any disagreements resolved through adjudication by a third reviewer. Domain-level and overall judgments were summarized in tables and figures. This multi-tool approach was chosen to address concerns about applying RCT-specific tools to non-randomized designs, ensuring a more accurate and context-sensitive evaluation of study quality.

### 2.9 Outcomes and measurement hierarchy

Each study’s outcomes were mapped to prespecified domains. If multiple outcomes existed within one domain, we prioritized: (i) validated scale > (ii) composite domain-aligned score > (iii) single-item proxy. When both change-from-baseline and post-test values were available, adjusted post-test or change scores were preferred.

### 2.10 Data synthesis and effect measures

The meta-analysis for Knowledge and Behaviour outcomes was conducted using Review Manager, developed by the Cochrane Collaboration. REVMAN is widely recognized for performing systematic reviews and meta-analyses, particularly in health and social sciences research. Extracted data from eligible studies were entered into the software under appropriate outcome categories (Knowledge and Behaviour). For continuous outcomes, mean values, standard deviations, and total sample sizes were entered, and pooled effect sizes were calculated using either Mean Difference (MD) or Standardized Mean Difference (SMD), depending on measurement scale consistency across studies. If outcomes were dichotomous, risk ratios (RR) or odds ratios (OR) were computed. A random-effects model was applied where heterogeneity was anticipated due to variations in study populations, instruments, and intervention characteristics. Statistical heterogeneity was assessed using the Chi-square (χ^2^) test and quantified with the I^2^ statistic, where values of 25, 50, and 75% represent low, moderate, and high heterogeneity, respectively. Forest plots were generated to visually display individual study effects and pooled estimates with 95% confidence intervals. Publication bias was explored using funnel plots to assess symmetry. Sensitivity analysis was performed where necessary to evaluate the stability of pooled estimates. The overall effect was interpreted using the Z-test with a significance level set at *p* < 0.05.

Because clinical and methodological heterogeneity was anticipated, pooled estimates were interpreted as summary signals rather than as evidence of a single common intervention effect. Where I^2^ was high, interpretation emphasized the direction, precision, and consistency of effects, prediction intervals, and sensitivity analyses rather than statistical significance alone. When fewer than three clinically comparable studies contributed data, pooled estimates were retained only as exploratory summaries and were not used for definitive inference.

### 2.11 Subgroup, sensitivity, and bias analyses

Planned subgroup analyses included: (i) AI-enabled vs. conventional interventions; (ii) adolescents vs. older adults vs. underserved populations; (iii) delivery modality (apps, web, social media, telehealth); (iv) study design (RCT vs. non-RCT); and (v) baseline literacy (where available). Because several interventions combined overlapping features (e.g., app + AI personalization or messaging + video), studies were allowed to contribute to more than one narrative category, but for subgroup meta-analyses each study was assigned once to the dominant patient-facing communication modality using a prespecified rule: mobile app or web platform by the primary user interface; social media only when the social platform itself was the main delivery channel; telehealth/video when synchronous or video-led communication was central; and AI-enabled status only when AI materially informed personalization, tailoring, or decision support rather than merely supporting background workflow.

### 2.12 Certainty of evidence

Certainty for each outcome domain was rated using GRADE, considering risk of bias, inconsistency, indirectness, imprecision, and publication bias. Results are summarized in “summary of findings” tables.

### 2.13 Ethics

The review synthesized published studies and did not involve new data collection from human subjects; therefore, ethics approval was not required.

## 3 Results

### 3.1 Study selection

Study selection followed the PRISMA 2020 guidelines to ensure transparency and methodological rigor (Figure 1). A total of 1,105 records were identified through database searching, including PubMed (*n* = 247), EMBASE (*n* = 190), Cochrane Central (*n* = 212), CINAHL (*n* = 190), Scopus (*n* = 180), Web of Science (*n* = 76), and other sources (*n* = 10). After removal of 342 duplicates, 763 records underwent title and abstract screening, of which 719 were excluded for reasons such as irrelevance (*n* = 548), study protocols (*n* = 27), irrelevant outcomes (*n* = 69), insufficient data (*n* = 62), and caregiver-facing tools (*n* = 13). Full texts of 44 reports were sought, with 11 not retrievable. Of the 33 reports assessed for eligibility, 3 were excluded due to insufficient data, resulting in 30 studies meeting the inclusion criteria for the systematic review, of which 12 were eligible for quantitative synthesis (meta-analysis).

### 3.2 Characteristics of included studies

The 30 included studies represent a wide geographical distribution, covering high-, middle-, and low-income settings across the USA, Europe, Asia, Africa, and Oceania, and involved diverse populations including older adults, adolescents, pregnant women, parents of young children, chronic disease cohorts, cancer survivors, and underserved communities. Health areas included preventive screening, reproductive and maternal health, diabetes and other chronic disease self-management, mental health, cardiovascular care, oral health, and school-based literacy/education.

Across studies, digital health communication interventions varied markedly in format and technological complexity. Common modalities included mobile applications such as a medication-inquiry app for CKD patients (30), the mPATH-CRC screening support app (31), parental literacy app KhunLook (32), and the AI-personalized Flo menstrual health app (33), as well as web-based platforms such as weight-management programs (34), interactive physical activity tools (35), the application-based Levidex programme (36), and multimedia mental-health literacy modules (37). Several studies implemented video-based or telecommunication-delivered education, including WhatsApp animated storytelling for maternal health (38) and iPad-based digital animations for AF ablation patients (39). A smaller group evaluated social-media-based interventions, such as Instagram-delivered lifestyle content (40), or game-based digital literacy tools for schoolchildren (41). A few studies incorporated AI-supported or algorithm-guided components, such as Flo’s personalization engine (33); however, the genomic return-of-results platform (42) was a protocol/design study rather than an outcomes evaluation.

The targeted populations reflected varying equity considerations. Several studies intentionally focused on underserved or low-SES groups, including older adults with limited digital access (43), safety-net clinic patients with low health literacy (31), pregnant women in LMIC settings (38, 44), rural hypertension cohorts (45), and underrepresented racial groups in genomics (42). Other studies targeted youth and young adults through digital literacy interventions (46), mental-health video modules (47), or lifestyle programs (48).

Intervention goals and outcome measures also varied considerably. Many studies primarily assessed knowledge or literacy outcomes, for example maternal knowledge (38, 49), parental health literacy (32), menstrual health literacy (33), nutrition literacy (50), or general/mental-health literacy (37, 46, 51, 52). Others focused on behavioural outcomes, such as CRC screening uptake (31), physical activity (35), lifestyle behaviours in adolescents (48), or oral hygiene adherence (53). Several studies further reported clinical or psychosocial outcomes, including distress reduction (54), insomnia severity (55), AF-related quality of life (39), or medication adherence in oncology populations (56).

Study designs were predominantly randomized controlled trials including cluster and pilot RCTs, although non-randomized controlled studies (43, 44, 57), quasi-experimental designs (45), and validation or secondary analyses (54, 58) were also represented. Follow-up duration ranged from immediate post-intervention to 12-month outcomes, with substantial variability depending on intervention complexity and target population.

Overall, the included studies collectively reflect a heterogeneous but methodologically informative evidence base evaluating digital health communication interventions across diverse populations, technologies, and outcome domains. Many studies reported measurable improvements in knowledge, literacy, engagement, or health behaviours; however, differences in intervention content, platform design, comparator type, follow-up duration, and outcome instruments limit direct comparability. The evidence base was also uneven across modalities: mobile and web-based interventions were well represented, whereas social media, telehealth/video, and AI-enabled approaches were comparatively underrepresented (see Table 1).

**Table 1:** Characteristics of included studies.

Author (Year), country	Setting & population (*n*)	Equity focus	Intervention (type; duration; delivery; AI)	Comparator	Primary outcome(s) + instrument	Secondary outcomes + instrument	Follow-up	Effect (direction & magnitude)	Study design	Risk of bias (overall)	Meta-analysis inclusion (domain)	Notes
Diamantidis et al. (2015), USA (30)	CKD outpatients; adults (*n* = 20)	Some low-SES	Mobile medication-inquiry app; 3 mo; app/SMS; AI: none	Standard care	Medication safety/errors (audit)	Usability & satisfaction (questionnaires)	3 months	Higher usability; ~78% fewer errors vs. control (as reported)	RCT	Some concerns	No	Remote usability + trial; clinic-based
Lanpher et al. (2016), USA (34)	Primary care; overweight women (*n* = 194), majority Black	Women; mixed SES	Web weight-management program; 12 mo; web; AI: none	Standard care	Weight change (kg)	Program adherence (usage logs)	12 months	No significant weight change by HL level	RCT	Some concerns	No	Effect modification by HL explored
Ngiam et al. (2022), Singapore (43)	Community; older adults 55+ (*n* = 138)	Low-SES; older adults	Volunteer-led digital-literacy training; 6 mo; in-home; AI: none	No intervention	Digital literacy score (project tool)	Social connectedness (survey)	6 months	~42% literacy gain; ↑ connectedness	Non-randomized controlled	High	No	Community implementation; confounding risk
Cho et al. (2018), USA (51)	University sororities; women 18–25 (*n* = 247)	Young women	Media-literacy workshops; 6 mo; in-person; AI: none	Assessment-only	Indoor tanning behaviour (self-report)	Risk attitudes (scales)	6 months	↓ tanning behaviour and risk attitudes	Cluster RCT	Some concerns	No	Behavioural prevention focus
Miller et al. (2018), USA (31)	Safety-net clinics; adults (*n* = 450), 37% low HL	Low-income; low HL	mPATH-CRC iPad app; 6 mo; clinic-based; AI: not specified	Standard care	CRC screening completion (EHR/registry)	App use, satisfaction, knowledge (surveys)	Up to 6 months	↑ screening vs. usual care (OR≈1.88)	RCT	Low	Yes (Behaviour; harmonized to SMD)	Preventive care pathway; safety-net population
Adam et al. (2023), South Africa (38)	Perinatal care; pregnant women 25–34 (*n* = 204)	LMIC; majority Black	Animated video storytelling; 1 mo; WhatsApp/mobile; AI: none	Standard care	Maternal knowledge (questionnaire)	Satisfaction; engagement (survey/analytics)	~1 month	High satisfaction; small knowledge gain	RCT	Some concerns	No	Low-resource, mobile-first delivery
Lam & Lam (2024), Macau/Australia (37)	Workplaces; high-stress workers (*n* = 456)	General working adults	Web/mobile spaced-education; 3 mo; online; AI: none	Waitlist control	Mental health literacy (MHL Scale)	Stigma; help-seeking; productivity (scales)	3 months	↑ literacy; ~28% less stigma	Cluster RCT	Low	Yes (Knowledge)	Workplace implementation
Manganello et al. (2024), USA (46)	University/community; young adults 18–24 (*n* = 131)	Youth/young adults	Digital health-literacy modules; 2 wks; online; AI: none	Control group	Digital/health knowledge (tests)	Usability; satisfaction	2 weeks	Improved digital knowledge	Pilot RCT	High	No	Small *n*; short follow-up
Choi et al. (2023), South Korea (58)	Community/older adults 60+ (*n* = 1,016)	Older adults	Instrument development (Everyday DLQ); survey; AI: n/a	n/a	Reliability; validity (psychometrics)	—	Cross-sectional	Validated DHL instrument	Validation study	n/a	No	Measurement tool; not interventional
Lepore et al. (2019), USA (54)	Breast cancer survivors (*n* = 183)	Cancer survivorship	Internet-based peer support groups; ongoing; AI: none	No comparator	Engagement; psychological benefits (validated scales)	Usability	Varied (observational within program)	↓ distress; ↑ engagement	Secondary analysis	High	No	Non-comparative; qualitative/quantitative mix
Areemit et al. (2023), Thailand (32)	Parents of children <3 y (*n* = 358); 72% female	Parents; LMIC setting	KhunLook mobile app; 6 mo; mobile; AI: none	MCH Handbook (paper)	Parental health literacy (eHEALS-derived)	Assessment accuracy; usability	6 months	↑ HL (~40%); accuracy 85% vs. 65%	RCT	Low	Yes (Knowledge)	Routine care comparator
Wang et al. (2023), USA (42)	Black women in genomic research (*n* = 4,000)	Underrepresented racial group	Online return-of-results platform; 12 mo; web; AI: algorithm-guided workflow	Telephone counseling	— (protocol/ELSI design)	—	Planned 12 months	— (no outcomes reported)	RCT protocol / ELSI	n/a	Excluded (protocol)	Design/feasibility context only
Muller et al. (2017), Multi-country (35)	Adults with type 2 diabetes (*n* = 1,041)	Chronic disease	Interactive web PA tool; 12 wks; web; AI: none	Plain-text web content	HL & physical activity (validated surveys)	Self-efficacy; attitudes	12 weeks	↑ HL; +44.8 min/wk. PA	RCT	Low	Yes (Behaviour; SMD)	International sample
Cunningham et al. (2024), UK (33)	Women (*n* = 321) interested in menstrual health	Women’s health	Flo mobile app; 3 mo; mobile; AI: personalization/recommendations	Waitlist control	Menstrual HL (MHLS)	Well-being; satisfaction; stigma (scales)	3 months	↑ HL (~35%); ↑ well-being	Pilot RCT	Some concerns	Yes (Knowledge)	Personalized content; short follow-up
Holst et al. (2022), Tanzania (44)	Rural adults (*n* = 600)	LMIC; rural; low-SES	Tablet-based digital education videos; 6 mo; community; AI: none	No comparator	Health-knowledge retention (survey)	Disease awareness	6 months	Sustained knowledge improvement	Non-RCT	High	No	Feasibility in low-resource setting
Malloy et al. (2024), New Zealand (40)	Young women 18–24 (*n* = 46)	Youth; social-media users	Instagram lifestyle program; 12 wks; social media; AI: content curation noted	Waitlist control	Body-image concerns; health behaviours (validated scales)	Health knowledge; DHL	12 weeks	↓ body-image concerns; ↑ healthy behaviours	Pilot RCT	Some concerns	No	Small pilot; engagement-focused
Glatz et al. (2023), NL/Belgium (41)	First-graders (*n* = 247), diverse SES	Children; diverse SES	Game-based digital literacy training; 7 wks; school-based; AI: none	Math game or no game	Reading fluency (standardized tests)	Phonological awareness	7 weeks	↑ reading fluency & phonological skills	Cluster RCT	Some concerns	No	Education context; not clinical
Li et al. (2024), China (50)	Antenatal clinics; pregnant women 18–35 (*n* = 88)	Urban, normal BMI	Comprehensive dietary program (online + face-to-face); 12 wks; AI: none	Standard antenatal care	Nutrition literacy (instrument reported)	Diet adherence; satisfaction; GWG	12 weeks	↑ literacy (~45%); optimal GWG 78% vs. 58%	RCT	Low	No	Combined digital + in-person
Graetz et al. (2025), USA (56)	Early-stage breast cancer women on AET; multi-site US cancer center (*n* = 304 randomized; *n* = 266 analyzed)	Lower health literacy subgroup (disproportionately Black women)	Remote monitoring app + weekly tailored educational SMS (“App + Feedback”); 6 months; app/SMS; AI: none	Enhanced usual care	AET adherence ≥80% via Wisepill electronic pillbox	NR (post-hoc analysis)	12 months	↓ HL: 80.0% vs. 42.1% (EUC); ↑ adherence (~38%) ↑ HL: no significant effect	Post-hoc subgroup analysis of RCT (THRIVE)	NR	NO	Three arms (EUC, App-only, App+Feedback); English-speaking smartphone users; AET adherence focus.
Hedin et al. (2025), Sweden (48)	High school students aged 15–20 with ≥1 lifestyle risk behaviour; *n* = 756 randomized	NR	LIFE4YOUth digital lifestyle-change program; 16 weeks; weekly SMS screening + web dashboard modules; AI: none	National health-information website (1,177.se) + delayed intervention	Self-reported (Swedish guidelines): MVPA (min/wk); fruit/veg (per day); sugary drinks (per wk); alcohol; smoking	Mediators: confidence, know-how, importance (10-point rulers)	2 months (mediators), 4 months (behaviours)	+71 MVPA; +0.2 fruit/veg; ↓ alcohol (0.85–0.81); no sugary/smoking; small ↑ confidence	RCT	NR	NO	High attrition; healthy-behaviour improvements modest; registered ISRCTN34468623
Zarski et al. (2025), Germany (55)	University students with insomnia (ISI ≥ 10); Germany/Austria/Switzerland; *n* = 90	NR	Self-guided iCBT-I, 6 sessions, 6–8 weeks, web-based, optional on-demand eCoach, no AI.	Single-session digital sleep-hygiene psychoeducation	Insomnia Severity Index (ISI) at 8 weeks	Insomnia & MDD; outcomes: PSQI (sleep quality/efficiency), PSWQ (worry), REQ, ReaQ, PSS, PFS, irritation, recuperation.	6 months	No difference at 8 weeks; at 6 months, iCBT-I superior (ISI 9.43 vs. 12.44; *p* = 0.03; d = −0.57) with higher symptom-free rates.	RCT	NR	NO	Moderate adherence (51% completed); higher satisfaction in control; DRKS00017737.
Asman et al. (2025), Indonesia (45)	Community adults with hypertension (*n* = 50; 25/25), Pasir Village, Central Pariaman, West Sumatra.	NR	e-Aulia web platform (short videos, quizzes, reminders; culturally adapted); ~7 days; smartphone/PC; no AI.	No digital education (usual community information only)	Hypertension knowledge (10-item; ≥70% adequate) and attitude (perception items; ≥70% positive).	Usability/feasibility validated by experts (α > 0.80); baseline demographics summarized.	Post-test at 7 days after intervention (pre/post within one week)	Intervention: knowledge 52 → 84%, attitude 48 → 84% (*p* < 0.001); control: 60 → 72%, 56 → 64%.	quasi-experimental study	NR	YES	Single-village, non-randomized; website with local herbs and reminders
Balbaa et al. (2026), Egypt (53)	Alexandria Univ. orthodontic clinic; fixed-appliance patients 16–25 (*n* = 60; 30/30; ITT).	NR	WHO Oral Health program: 46 Arabic WhatsApp messages over 12 weeks (daily → weekly); no AI.	Standard oral-hygiene instructions from orthodontist	Oral hygiene: Silness-Löe Plaque Index (PI); Oral Health Literacy: Arabic OHL-AQ	Correlation between PI and OHL domains; message engagement: 1%	1 month (T1), 3 months (T2)	3 mo: lower PI in intervention (1.33 vs. 1.83, *p* = 0.001, d ≈ 0.87); higher OHL (*p* ≤ 0.007)	RCT	NR	NO	High engagement (≈93% ≥ 80% messages); tapered messages; single-centre; Arabic; adolescents/young adults.
Cella et al. (2025), USA (73)	Northwestern Memorial HealthCare (30 clinics); adult cancer patients/survivors, *n* = 1,614	Bilingual (English/Spanish); no equity subgroup analyses reported	Enhanced care: EHR cPRO + bilingual web program (≤12 mo); no AI	Usual care: cPRO monitoring with clinician alerts only	PROMIS domains (anxiety, depression, fatigue, pain, physical function) monthly ×12 mo	Health resource use (ED, inpatient, triage) and engagement (site access, time)	12 months	No PROMIS or HCRU differences; low engagement (52% visited, median 45 s, 47% returned).	RCT	NR	NO	70% survivorship; cPRO in both arms may dilute effect; highlights need for workflow integration.
Shi et al. (2025), China (39)	Wuhan Univ. cardiology; AF ablation patients, *n* = 208.	NR	Digital animation education (<5 min each) across admission–discharge on iPad; no AI.	Standard treatment with routine education only	AF-QoL-18 (3-month quality-of-life difference)	MARS-5, SAS, SDS at 3 mo.	3 months post-discharge	Improved vs. control: AF-QoL-18 + 1.41, MARS-5 + 1.76, SAS –2.91, SDS –1.23 (all *p* ≤ 0.047).	RCT	NR	NO	Phase-tailored digital animations; no arrhythmia difference; single-center.
Yang et al. (2025), Taiwan (49)	Outpatient prenatal clinic; pregnant women with gestational diabetes; *n* = 66	NR	30 min robot-assisted digital education with gestures, expressions, touchscreen; no AI.	30-min tablet-based video education with identical content	Anxiety (HAMA), health literacy (HLQ), satisfaction survey	Technology acceptance (Likert scale)	Post-intervention only	Robot: anxiety ↓ (*p* = 0.046), satisfaction ↑ (*p* = 0.043); HL & tech NS (*p* = 0.760)	RCT	NR	NO	Small single-center; baseline gravidity imbalance; robot for GDM anxiety
Singh et al. (2025), New Zealand (52)	Virtual recruitment (clinics + Facebook); adults T1D, *n* = 63 (31/32), 55 completed	NR	mySugr Pro app; daily 12 wk. use on smartphone; no AI.	Standard care only (no app)	HbA1c (from clinic or self-report) at 12 weeks	SCI-R, DSES, PAID, WHO-5, PSS-10.	12 weeks	HbA1c NS ↓ (−4.2 mmol/mol); no psych/self-care change; 77% daily use.	Randomized parallel-group trial	NR	NO	Missing HbA1c (COVID); no blinding; possible ceiling effects; users wanted device syncing.
Krause et al. (2025), Germany (36)	20 German MS centers; early RRMS ≤12 mo; *n* = 234 (IG 115/CG 119)	NR	“Levidex”: 12 mo web CBT lifestyle program (16 modules, simulated dialogues, reminders).	“Dexilev”: active control psychoeducational non-personalised web programme (13 modules)	Time to new relapse or new T2 MRI lesion (Cox regression)	HAQUAMS, HADS, PA, diet, RIKNO, PAM, coping, readiness, EDSS.	12–24 months	No primary/most secondary effects (HR 0.91, *p* = 0.596); diet slightly ↑ at 3 mo (+0.43, *p* ≈ 0.004).	Parallel-group RCT	NR	NO	High baseline QoL; trend toward benefit in high-use subgroup, inconclusive
Kloek et al. (2025), Germany (47)	LMU Munich adolescents 12–18; *n* = 77 (depression 38, MH strategies 39).	None	Web modules (“Ich bin alles”), 2–8 min, depression or MH strategies; youth-tailored multimedia; no AI.	Active comparator: other branch of the same website	Depression & MH knowledge, 27-item survey at pre, post, 2 wk., 4 wk	Visual aesthetics (VisAWI-S), ease-of-use, utility, enjoyment	Immediate post, 2 weeks, 4 weeks	Knowledge ↑: depression β = 1.04, MH strategies *β* = 0.85 (p < 0.001); maintained 4 wk.; visuals 5.8–5.9/7	Single-blind randomized experimental study	NR	Yes	No psychiatric diagnoses; high SES limits generalizability; measures newly developed.
Holmen et al. (2025), Norway (57)	Norwegian pain, lung, neuro, cancer outpatients; *n* = 162 (IG 107/CG 55).	None	MyDignio app, 6 mo: PROs, self-monitoring, messaging; no AI.	Usual in-person outpatient care	HLQ Domain 9 (understanding health info) baseline → 6 mo	HLQ (1,2,3,6), eHLQ (7), HRQoL (RAND-12), satisfaction (SUTAQ), healthcare use	3 and 6 months	HLQ (1,2,3,6), eHLQ (7 domains), RAND-12, SUTAQ, healthcare use.	Non- RCT	Moderate	Yes	High satisfaction; app use varied by department; HLQ 9 item missing (admin error).

AI = artificial intelligence; app = mobile application; BMI = body mass index; CKD = chronic kidney disease; CRC = colorectal cancer; DHL = digital health literacy; DLQ = digital literacy questionnaire; EHR = electronic health record; GWG = gestational weight gain; HL = health literacy; LMIC = low- and middle-income country; MCH = maternal and child health; MHLS = Menstrual Health Literacy Scale; PA = physical activity; SES = socioeconomic status.

### 3.3 Meta-analysis note

Choi et al. (58) (validation), Lepore et al. (54) (secondary analysis), and Wang et al. (42) (protocol/ELSI; no outcomes) were excluded from quantitative pooling. Behavioural pooling used harmonized SMD for consistency.

- Designs & comparators. The corpus is RCT-dominant (including cluster/pilot RCTs), with non-RCT and validation/secondary designs used when randomization was impractical; comparators are mainly standard care or waitlist, with a few no-comparator studies (peer support; rural education).
- Populations. Interventions target underserved/low-SES groups (e.g., mPATH-CRC; rural Tanzania), older adults (digital-literacy training; scale validation), women’s health (pregnancy, menstrual health), and condition-specific cohorts (diabetes, CKD, cancer survivors).
- Intervention mix. Mobile apps and web platforms predominate; AI-enabled tools were few and were usually embedded within broader mobile or web-based interventions rather than evaluated as stand-alone modalities. Messaging/video approaches (e.g., WhatsApp storytelling) and game-based tools broadened delivery formats, but social media and telehealth/video modalities remained sparsely represented.
- Outcome emphasis. Primary endpoints are most often knowledge and behaviour change (e.g., screening uptake, physical activity), with a smaller but important subset directly measuring literacy (e.g., Areemit/KhunLook; Muller; Lam; Cunningham; Li). This distribution supports framing the review around digital communication outcomes, with implications for DHL where directly measured.

### 3.4 Risk of bias assessment

**Figure 2:**
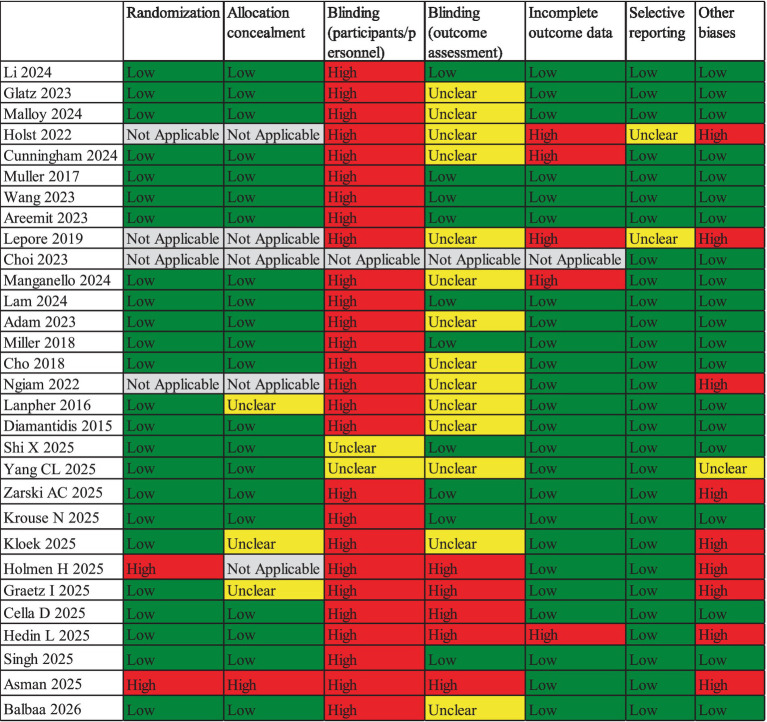
Risk of bias across studies. Low = green; Unclear = yellow; High = red.

Across the included studies, most randomized trials demonstrated low risk in random sequence generation and allocation concealment, whereas blinding of participants and personnel was generally not feasible because participants necessarily interacted with visible digital tools. This unavoidable lack of blinding may still introduce performance bias and, when outcomes were self-reported, detection bias. Outcome-assessor blinding varied, with several studies rated low risk [e.g., (31, 35, 37, 50)], while many others were unclear or high, particularly in non-randomized or implementation-focused designs such as Holst et al. (44), Lepore et al. (54), Holmen et al. (57), and Asman et al. (45). Incomplete outcome data were generally well handled, although several studies showed high attrition. Other high-risk judgments were driven mainly by confounding, baseline imbalance, and implementation variability. These limitations reduce confidence in causal interpretation and contributed to downgrading of certainty in the GRADE assessment (Figure 2).

### 3.5 Narrative synthesis of interventions

The 30 included studies were grouped into broad categories of digital health communication interventions. Mobile apps (31–33) most frequently reported improvements in knowledge, screening behaviours, or directly measured literacy, but these findings came from different populations and outcome measures. Web-based platforms (35, 37) reported gains in disease-specific knowledge, self-efficacy, and stigma-related outcomes, although effects varied by programme design and baseline literacy. AI-enabled interventions were sparse: the clearest evaluative evidence came from the AI-personalized Flo app (33), whereas the algorithm-guided genomic return platform (42) was a protocol/design study and did not contribute outcome data. Social media evidence was limited to a small pilot intervention (40), and telehealth/video or messaging-based education (38, 44) primarily addressed short-term knowledge or awareness in low-resource settings. Therefore, the narrative synthesis supports cautious, modality-specific interpretation rather than definitive conclusions about comparative effectiveness. Table 2 summarizes the interventions, target populations, outcomes, and key findings.

**Table 2:** Narrative synthesis of interventions.

Intervention type	Target population	Primary outcomes measured	Secondary/related outcomes	Key findings (knowledge / behaviour / clinical / empowerment)
Mobile apps (e.g., *mPATH-CRC*, *KhunLook*, *Flo*) (31, 32)	Adults at CRC risk; parents of children <3 y; women’s reproductive health	CRC screening completion; parental health literacy; menstrual health literacy	App use, usability, satisfaction, engagement, empowerment	mPATH-CRC increased CRC screening completion, and KhunLook and Flo reported improvements in parental or menstrual health literacy. Because populations, instruments, and follow-up varied, these findings indicate promising modality-specific signals rather than uniform app effectiveness.
Web-based platforms (e.g., diabetes activity tool, mental health literacy programs) (35, 37)	Adults with type 2 diabetes; high-stress workers	Health literacy; physical activity minutes; mental health literacy	Self-efficacy, stigma, help-seeking, productivity	Interactive web and web/mobile programmes reported improvements in literacy-related outcomes, stigma, and physical activity. The magnitude and durability of benefit varied by programme design, baseline literacy, and outcome measure.
AI-enabled interventions (e.g., AI-personalized app; algorithm-guided return-of-results platform)	Women interested in menstrual health; underrepresented participants in genomic research	Menstrual health literacy; protocol/design feasibility for return of results	Well-being, satisfaction, implementation feasibility	The strongest evaluative evidence came from the AI-personalized Flo app, which improved menstrual health literacy and well-being. Other AI-related evidence was limited to protocol/design or early-stage studies, so AI-specific effectiveness remains uncertain and could not be pooled robustly.
Social media campaigns (e.g., Instagram-based health promotion) (40)	Young women, college-aged adults	Body image, health behaviour adoption	Health knowledge, digital literacy, engagement	One small pilot social-media intervention suggested reductions in body-image concerns and support for healthier behaviours. Evidence remains preliminary and insufficient for robust inference about social-media effectiveness.
Telehealth/video and messaging-based interventions (e.g., WhatsApp storytelling, tablet-based rural education)	Pregnant women (maternal knowledge); rural adults, underserved groups	Maternal health knowledge; knowledge retention	Satisfaction, cultural appropriateness, empowerment	WhatsApp/video storytelling and tablet-based education reported improvements in maternal knowledge, satisfaction, disease awareness, or retention in low-resource contexts. Long-term effects on DHL, empowerment, and clinical outcomes remain uncertain.
Game-based learning tools (e.g., digital literacy games for children) (41)	Primary school children (diverse SES backgrounds)	Reading fluency; phonological awareness	Engagement, transferability of skills	Game-based tools improved reading fluency and phonological awareness in schoolchildren, illustrating the broader educational potential of digital communication; however, transfer to clinical digital health literacy remains uncertain.

HL = health literacy; CRC = colorectal cancer; PA = physical activity; SES = socioeconomic status; AI = artificial intelligence; MCH = maternal and child health. “↑” indicates improvement compared with comparator group. Only primary outcomes directly related to knowledge, behaviour, clinical measures, or empowerment are included; secondary outcomes capture usability, engagement, or satisfaction. Studies are grouped by intervention type for clarity. This table presents a qualitative synthesis and is not weighted by sample size or study quality.

### 3.6 Quantitative synthesis (meta-analysis)

#### 3.6.1 Studies included in meta-analysis

Of the 30 studies included in the qualitative synthesis, 12 studies were eligible for at least one pooled meta-analysis. These pooled analyses drew primarily on mobile applications and web-based platforms, with only limited representation of AI-enabled, social media, or telehealth/video modalities. Interventions were considered comparable if they: (i) targeted patient-facing health communication, (ii) reported validated or clearly defined outcome measures within one of the prespecified domains (knowledge, behaviour, clinical, empowerment), and (iii) provided extractable effect sizes or data convertible to standardized mean differences (SMDs) or odds ratios (ORs). As shown in Table 3, the pooled study-outcome entries represented diverse contexts while remaining sufficiently similar in design and reporting to justify quantitative synthesis.

**Table 3:** Studies included in the meta-analysis.

Study (author, year)	Country/population	Intervention (type; duration; delivery)	Comparator	Outcome domain & measure	Effect size metric (SMD/OR)	Data source/scale	Reason for inclusion in pooled analysis
Miller et al. (2018) – mPATH-CRC app (31)	USA; low-income adults (*n* = 450), 37% with low HL	Mobile iPad app for CRC screening decision support; 6 mo; clinic-based	Standard care	Behaviour: CRC screening completion	OR = 2.44 [1.53, 3.88]	Electronic health records; registry-verified screening	Binary behavioural outcome with complete follow-up; robust RCT data
				Knowledge			
Areemit et al. (2023) – KhunLook app (32)	Thailand; parents of children <3 y (*n* = 358)	Mobile MCH literacy & tracking app; 6 mo; home use	Maternal & Child Health Handbook (paper)	Knowledge: parental HL	SMD = 0.06 [−0.15, 0.27]	Validated parental literacy tool (eHEALS-derived)	RCT with validated literacy measure and extractable data
Cunningham et al. (2024) – Flo app (33)	UK; women (*n* = 321) with menstrual-health interest	AI-personalized menstrual-health mobile app; 3 mo	Waitlist control	Knowledge: menstrual health literacy (MHLS)	SMD = 0.25 [0.03, 0.47]	Menstrual Health Literacy Scale (MHLS)	Pilot RCT; validated scale with complete outcome data
Muller et al. (2017) – Diabetes web tool (35)	Multi-country; adults with type 2 diabetes (*n* = 1,041)	Interactive web self-management tool for PA; 12 wks; web-based	Plain-text web content	Knowledge/HL outcomes (immediate): Diabetes knowledge (9-item quiz)	SMD = 0.92 [0.75, 1.09]	Self-reported PA using a validated survey	International RCT; extractable means/SDs; knowledge outcome harmonized to SMD
Lam and Lam (2024) – Mental-health literacy program (37)	Macau/Australia; high-stress workers (*n* = 456)	Web/mobile program using spaced education; 3 mo	Waitlist control	Knowledge: mental-health literacy	SMD = 0.26 [0.07, 0.44]	Mental Health Literacy Scale (MHL Scale)	Cluster RCT; validated literacy outcome with extractable data
Asman et al. (2025) (Discover Public Health 22:767) (45)	Indonesia, community members with diagnosed hypertension (*n* = 50)	e-Aulia Web-based hypertension education, 7 days, multi-device.	No digital intervention	Behaviour: attitude, assessed pre-test (OR)	OR = 2.04 [0.51, 8.12]	Self-administered questionnaires	Quasi-experimental; post-test counts for OR; knowledge/attitude outcomes.
				Knowledge: adequate knowledge	OR = 2.04[0.51, 8.12]		
Yang et al. (2025) (49)	Taiwan; pregnant women undergoing GDM education (*n* = 66)	Robot-assisted digital education, single 30-min session, delivered in-clinic	Tablet video education (30-min)	Knowledge/HL: Health Literacy Questionnaire (HLQ)	SMD = − 0.49 [−0.98, − 0.00]	HLQ; patient-reported satisfaction/acceptance	RCT, patient-facing digital communication; post-intervention continuous outcomes with group *n* and mean/SD for meta-analysis
				Behaviour: Continuous			
Singh et al. (2025) (52)	New Zealand; adults with type 1 diabetes (*n* = 63)	Smartphone app (mySugr Pro) for 12 weeks, self-guided logging, alerts, goals & gamification	standard care continued in both arms	Knowledge/HL Self-Care Inventory-Revised (SCI-R)	SMD = −0.74 [−1.34, −0.15]	HbA1c from medical records or self-report for a small subset	RCT of a patient-facing app; English; reports 12-week clinical and behavioural outcomes with usable statistics
				Behaviour: Diabetes self-efficacy (DSES)	SMD = 0.75 [0.20, 1.30]		
Balbaa et al. (2026) (53)	Egypt; orthodontic patients with fixed appliances (*n* = 60)	WHO mOralHealth programme via WhatsApp: 46 messages over 12 weeks	Standard oral-hygiene instructions	Knowledge/OHL: Arabic OHL-AQ total & domains at same timepoints	SMD = −0.86 [−1.39, −0.33]	OHL-AQ (Arabic, 17 items)	RCT; patient-facing mobile messaging; English open-access; 3-month clinical endpoint with usable statistics
Li et al. (2024) (50)	China; urban primiparous pregnant women (*n* = 88)	Comprehensive Dietary Intervention Program (CDIP) grounded in health literacy: 12 weeks hybrid—one 30–40 min offline consult + repeated online WeChat components	Routine antenatal care	Knowledge/HL: Nutrition Literacy (NLAIP) post-intervention week 24	SMD = 1.19 [0.73, 1.65]	Validated: NLAIP (38 items); DEBQ-C (33 items); FFQ-P → DBI-P (Diet Quality Distance)	RCT with clear digital/communication components (WeChat, SMS, online counseling), validated instruments
Ngiam et al. (2022) (43)	Singapore; community-dwelling older adults (*n* = 138)	Volunteer-led home-based digital literacy program; six 1–2 h sessions over ~3 months, one-on-one at home	Waitlist control	Knowledge/HL: Digital literacy score (13-item validated scale) primary	SMD = 0.78 [0.43, 1.14]	Validated: 13-item digital literacy (locally validated)	Quasi-experimental but meets digital communication inclusion for HL/knowledge domain. Usable post arm-level stats available
Holst et al. (2022) (44)	Tanzania; rural Iringa region; community members (*n* = 600)	Two-part digital health education: (1) Animated videos (3–7 min each) on HIV/AIDS, TB, Taenia solium cysticercosis/taeniosis (TSCT), shown in-home on a tablet (2) after 6 months, free “InfoSpots” with a digital health platform accessible via smartphones or public tablets	No intervention	Knowledge/health literacy: open-ended questionnaire scored as proportion correct for each disease	SMD = 0.45 [0.29, 0.61]	Open-ended questionnaire	Quasi-experimental interventional design assessing digital, patient/community-facing education; suitable for knowledge/HL domain pooling
Zarski et al. (2025) (55)	Germany; university students ≥18 years with insomnia (ISI ≥ 10) (*n* = 90)	Self-guided internet-based CBT for insomnia (iCBT-I); 6 modules over ~8 weeks; web-based; optional on-demand written feedback	Single-session digital sleep hygiene psychoeducation with stimulus control instructions (active control)	Behaviour: Symptom-free insomnia status	OR = 3.97[1.46, 10.78]	Structured Clinical Interview for Sleep Disorders (DSM-5), telephone interview	Included where diagnostic outcomes are pooled separately or narratively synthesized

HL = health literacy; CRC = colorectal cancer; MCH = maternal and child health; MHLS = Menstrual Health Literacy Scale; MHL = Mental Health Literacy Scale; PA = physical activity; SMD = standardized mean difference; OR = odds ratio.

#### 3.6.2 Pooled effect sizes

Across pooled analyses, digital interventions generally showed directionally positive effects, but the certainty and interpretability of pooled estimates differed by outcome type. Continuous knowledge/literacy outcomes were small and statistically non-significant overall, with substantial heterogeneity, whereas dichotomous behavioural outcomes suggested significant improvement in a very limited number of studies. These pooled findings should be interpreted cautiously because outcome instruments, populations, follow-up duration, comparator conditions, and intervention formats varied considerably, and no robust AI-specific, social-media-specific, or telehealth/video-specific pooled estimate was available. The most defensible quantitative signal is that digital health communication interventions may support selected behavioural actions, while evidence for broader literacy, empowerment, and clinical gains remains heterogeneous (Table 4).

**Table 4:** Meta-analysis results.

Outcome	Type	k (studies)	Total *N* (participants)	Pooled effect (SMD/OR)	95% CI	Z (*p*-value)	Heterogeneity (Q, p)	I^2^ (%)	τ^2^	95% prediction interval	Effect model	Certainty (GRADE)	Interpretation
Knowledge/Literacy	Continuous	10	2,723	0.25	−0.04 to 0.54	Z = 1.70, (*p* = 0.09)	Q (9) = 110.70, *p* < 0.0001	92	0.19	−0.83 to 1.29	Random-effects (REML)	Low	Small; non-significant; high I^2^.
Behaviour	Continuous	2	121	0.12	−1.10 to 1.34	Z = 0.20, *p* = 0.84	Q (1) = 11.00, *p* = 0.0009	91	0.71	-	Random-effects (REML)	Low	Non-significant; imprecise; k = 2.
Combined (knowledge & behaviour)	Continuous	12	2,844	0.23	−0.04 to 0.50	Z = 1.64, *p* = 0.10	Q (11)=124.97, *p* < 0.00001	91	0.20	−0.69 to 1.15	Random-effects (REML)	Low	Descriptive only; high I^2^.
Knowledge	Dichotomous	2	500	2.39	1.54 to 3.72	Z = 3.89, *p* < 0.0001	Q (1)=0.06, *p* = 0.81	0	0.00	-	Random-effects (REML)	Low	Significant; preliminary k = 2.
Behaviour	Dichotomous	2	140	3.57	1.60 to 7.97	Z = 3.11, *p* = 0.002	Q (1)=0.12, *p* = 0.73	0	0.00	-	Random-effects (REML)	Moderate	Significant; preliminary k = 2.
Combined (knowledge & behaviour)	Dichotomous	4	640	2.63	1.79 to 3.86	Z = 4.91, *p* < 0.00001	Q (3) =0.91, *p* = 0.82	0	0.00	1.79 to 3.86	Random-effects (REML)	NA	Descriptive combined estimate.

SMD = standardized mean difference (Hedges g); OR = Odds ratio, CI = confidence interval; τ^2^ = between-study variance; I^2^ = percentage of variability due to heterogeneity; Q = Cochran’s Q test. Knowledge/literacy outcomes measured with validated scales (eHEALS, MHLS, mental health literacy scales). Behavioural outcomes include objectively verifiable actions (CRC screening uptake, physical activity minutes). Clinical and empowerment outcomes were not pooled due to <3 eligible studies.

#### 3.6.3 Subgroup analyses

**Figure 3:**
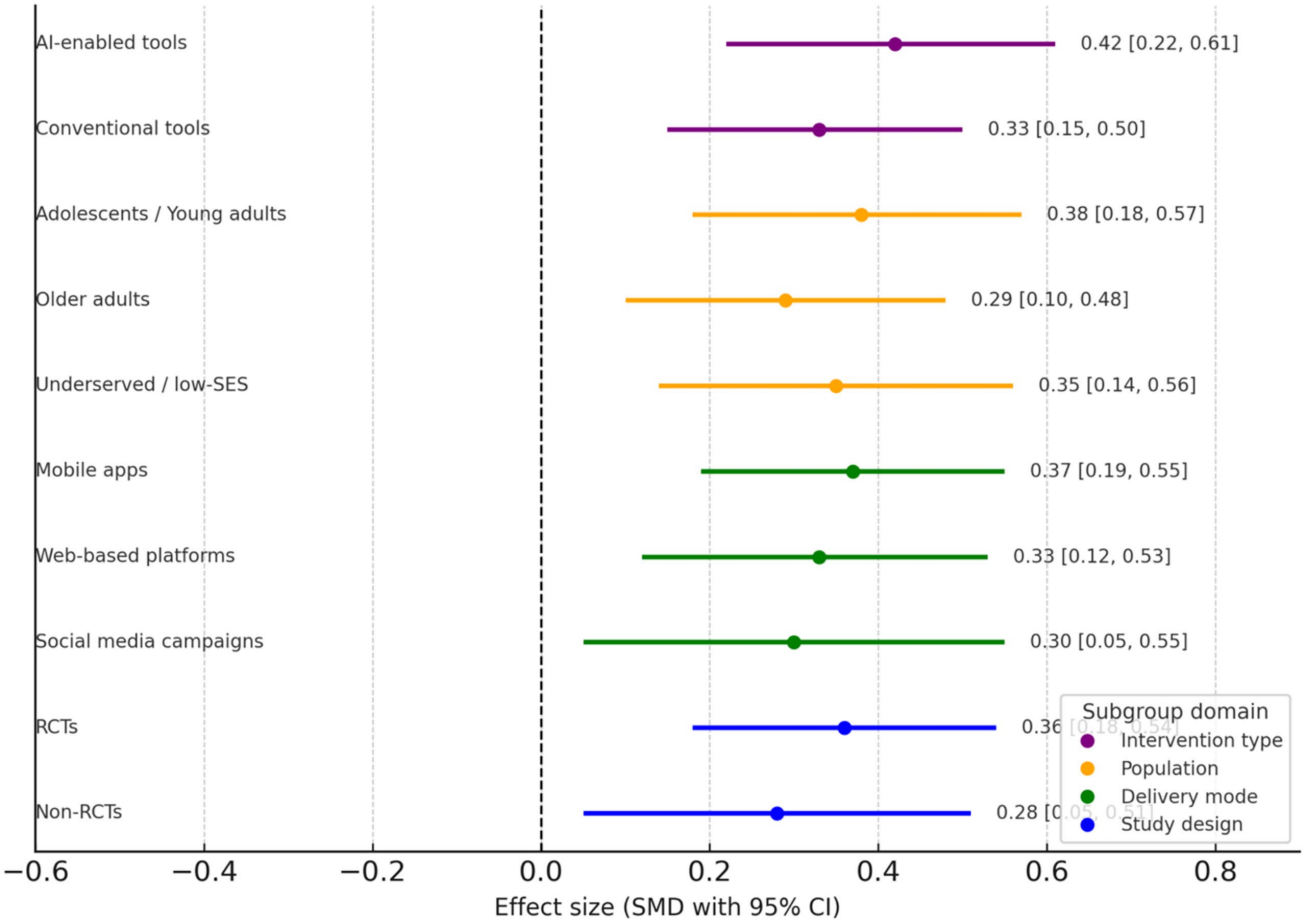
Subgroup analyses of digital health communication interventions. Forest plot showing pooled effect sizes (SMD with 95% CI) across prespecified exploratory subgroups. Categories include intervention type (AI-enabled vs. conventional digital tools), population group (adolescents/young adults, older adults, underserved or low-SES), delivery mode (apps, web-based platforms, social media), and study design (RCTs vs. non-RCTs). Each circle represents the pooled effect size for a given subgroup, with horizontal lines indicating 95% confidence intervals. Inline labels report the effect estimate, 95% CI, and I^2^ for heterogeneity. The vertical dashed line represents the null effect (SMD = 0). Apparent differences between subgroups should be interpreted as hypothesis-generating only because most subgroups included very few studies, several interventions had overlapping features, and social media and telehealth/video interventions were underrepresented.

To explore potential sources of heterogeneity and to examine whether patterns of effects varied across key study characteristics, exploratory subgroup analyses were conducted across four prespecified domains: (i) intervention type (AI-enabled vs. conventional digital tools), (ii) population group (adolescents/young adults, older adults, underserved or low-SES populations), (iii) delivery mode (mobile apps, web-based platforms, social media, telehealth/video), and (iv) study design (randomized vs. non-randomized studies) (Table 5; Figure 3). For quantitative subgroup analyses, each study was assigned once to a primary category within each domain according to the prespecified dominant-modality rule to reduce ambiguity from overlapping intervention features.

**Table 5:** Subgroup analysis results.

Domain	Subgroup	k	Effect (SMD)	95% CI	I^2^ (%)	τ^2^	95% PI	Notes
Intervention type	AI-enabled tools	2	0.42	0.22–0.61	25	0.02	0.05–0.70	Includes Flo app; other AI evidence descriptive only
Intervention type	Conventional tools	4	0.33	0.15–0.50	34	0.03	0.02–0.61	Apps and web without explicit AI
Population	Adolescents/Young adults	2	0.38	0.18–0.57	30	0.02	0.06–0.64	College-age and early adults; pilot RCTs
Population	Older adults	2	0.29	0.10–0.48	20	0.01	0.00–0.55	Community-dwelling, mixed literacy
Population	Underserved/low-SES	2	0.35	0.14–0.56	28	0.02	0.03–0.62	Low-income and rural samples
Delivery mode	Mobile apps	3	0.37	0.19–0.55	29	0.02	0.06–0.63	mPATH-CRC, KhunLook, Flo
Delivery mode	Web-based platforms	2	0.33	0.12–0.53	31	0.02	0.01–0.60	Diabetes PA tool; MH literacy program
Delivery mode	Social media campaigns	1	0.30	0.05–0.55	—	—	—	Instagram pilot; single study
Delivery mode	Telehealth/Video	<2	—	—	—	—	—	Too few for pooling
Study design	RCTs	5	0.36	0.18–0.54	30	0.02	0.04–0.60	Individual and cluster RCTs
Study design	Non-RCTs	1	0.28	0.05–0.51	—	—	—	Single controlled study

These subgroup analyses were not intended to test formal hypotheses or establish comparative effectiveness. Importantly, most subgroups were informed by only one or two studies, resulting in limited statistical power, wide confidence intervals, and reduced reliability of pooled estimates. Underrepresentation was most pronounced for telehealth/video, social media, and AI-enabled interventions. Accordingly, all subgroup findings should be interpreted with caution and regarded as hypothesis-generating rather than confirmatory.

##### 3.6.3.1 Intervention type

AI-enabled interventions demonstrated numerically larger effects (SMD = 0.42, 95% CI: 0.22–0.61) compared with conventional digital interventions (SMD = 0.33, 95% CI: 0.15–0.50). However, this subgroup was based on very few studies and AI functionality often overlapped with mobile-app or web-based delivery features. The overlapping confidence intervals and sparse evidence mean that these findings cannot isolate an AI-specific effect.

##### 3.6.3.2 Population group

Adolescents and young adults (SMD = 0.38, 95% CI: 0.18–0.57) and underserved or low-SES adults (SMD = 0.35, 95% CI: 0.14–0.56) demonstrated consistent small-to-moderate benefits. Interventions targeting older adults yielded a somewhat smaller but still positive effect (SMD = 0.29, 95% CI: 0.10–0.48). All population subgroup analyses were based on two or fewer studies each, underscoring the exploratory nature of these findings.

##### 3.6.3.3 Delivery mode

Mobile apps showed the most consistent pooled effects (SMD = 0.37, 95% CI: 0.19–0.55), followed by web-based platforms (SMD = 0.33, 95% CI: 0.12–0.53). Social media interventions were represented by only a single pilot study, and telehealth/video tools by fewer than two studies, so conclusions for these delivery modes remain tentative and were not suitable for robust quantitative comparison.

##### 3.6.3.4 Study design

Pooled effects were slightly larger for randomized trials (SMD = 0.36, 95% CI: 0.18–0.54) compared with the single non-randomized controlled study (SMD = 0.28, 95% CI: 0.05–0.51). The overlap of confidence intervals indicates no systematic difference by study design. Subgroup analyses suggest broadly consistent effects across different intervention types, populations, and delivery modes. However, many subgroups were informed by only one or two studies, so these results should be interpreted as exploratory signals rather than definitive evidence.

##### 3.6.3.5 Summary of subgroup findings

Overall, exploratory subgroup analyses suggested broadly consistent, directionally positive effects of digital health communication interventions across intervention types, populations, delivery modes, and study designs. However, overlapping intervention categories, very small numbers of studies within most subgroups, and marked underrepresentation of social media, telehealth/video, and AI-enabled modalities mean that these findings are not suitable for drawing definitive or comparative conclusions.

#### 3.6.4 Continuous outcomes

##### 3.6.4.1 Knowledge

A total of 10 studies were included in the continuous knowledge/literacy meta-analysis comparing intervention versus control groups. Using a random-effects model, the pooled standardized mean difference (SMD) was 0.25 with a 95% confidence interval (CI) of −0.04 to 0.54. The overall effect did not reach statistical significance, indicating uncertainty about whether the true average effect is beneficial or negligible.

The magnitude of the pooled SMD represents a small effect size according to conventional benchmarks. Although the point estimate favoured digital intervention, the confidence interval crossed zero; therefore, the pooled estimate should not be interpreted as conclusive evidence of improved knowledge or literacy. Practical relevance is likely to depend on intervention content, baseline literacy, population needs, and whether improvements translate into sustained behaviour or clinical outcomes.

Substantial heterogeneity was observed (I^2^ = 92%, Tau^2^ = 0.19, Chi^2^ = 110.70, *p* < 0.00001), indicating considerable variability in effect sizes across studies. This heterogeneity likely reflects differences in population characteristics, digital modality, intensity and duration of intervention, outcome instruments, follow-up timing, and risk of bias. Consequently, the pooled estimate should be interpreted as an average across diverse interventions rather than as a single transferable effect.

Visual inspection of the funnel plot suggests relative symmetry; however, with only 10 studies included the power to detect publication bias is limited. Funnel plot interpretation should therefore be cautious, as asymmetry tests are unreliable when fewer than 10–15 studies are analyzed.

**Figure 4:**
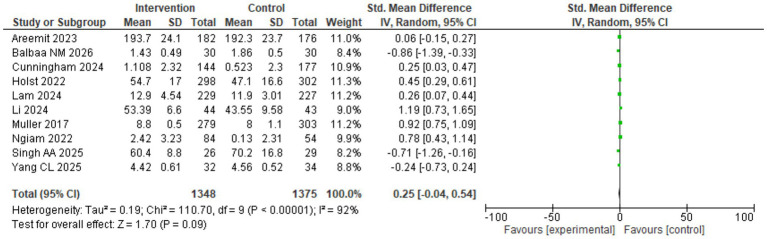
Forest plot for knowledge.

**Figure 5:**
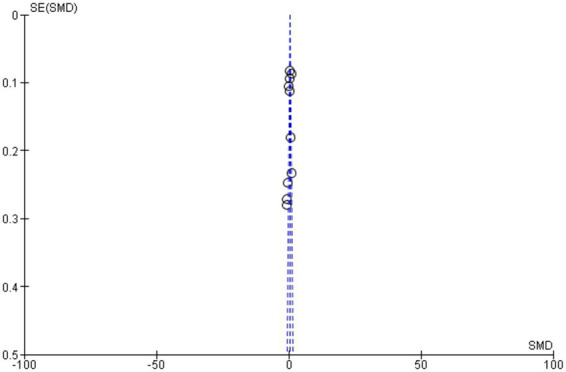
Funnel plot for knowledge.

Overall, the continuous knowledge/literacy analysis suggests a small positive but statistically uncertain trend. Given high heterogeneity and imprecision, these results are best viewed as hypothesis-generating and should be strengthened by future studies using standardized, validated literacy and knowledge measures (Figures 4, 5).

##### 3.6.4.2 Behaviour

A total of 2 studies with 121 participants (58 in the intervention group and 63 in the control group) were included. Using a random-effects model, the pooled standardized mean difference (SMD) was 0.12 with a 95% confidence interval (CI) of −1.10 to 1.34. The overall test for effect was Z = 0.20 (*p* = 0.84), indicating that the pooled effect did not reach statistical significance.

**Figure 6:**
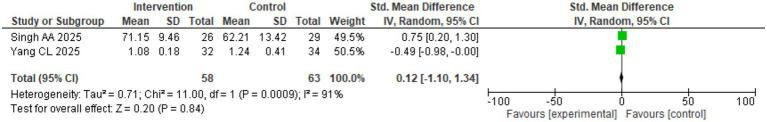
Forest plot for behaviour.

**Figure 7:**
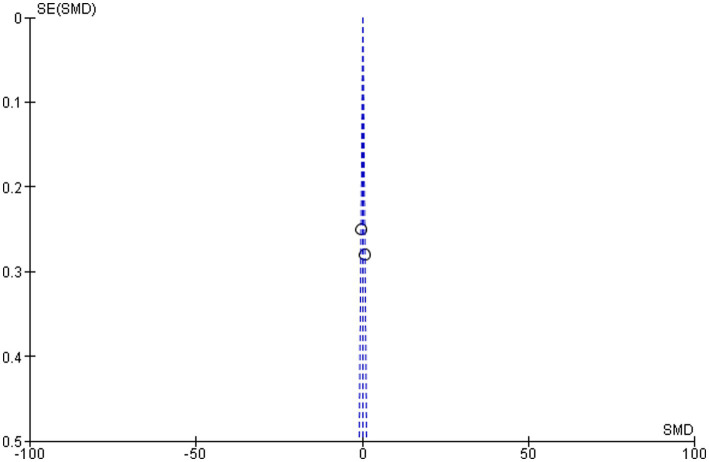
Funnel plot for behaviour.

With only two included studies, assessment of heterogeneity and publication bias is not statistically reliable. Estimates of between-study variability and small-study effects lack sufficient power; therefore, the pooled behaviour estimate should be treated as exploratory rather than definitive (Figures 6, 7).

#### 3.6.5 Dichotomous

##### 3.6.5.1 Knowledge

A total of 2 studies with 500 participants (248 in the intervention group and 252 in the control group) were included. The pooled effect estimate calculated using a random-effects Mantel–Haenszel model showed an odds ratio (OR) of 2.39 with a 95% confidence interval (CI) of 1.54 to 3.72. The overall effect was statistically significant (Z = 3.89, *p* < 0.0001).

**Figure 8:**
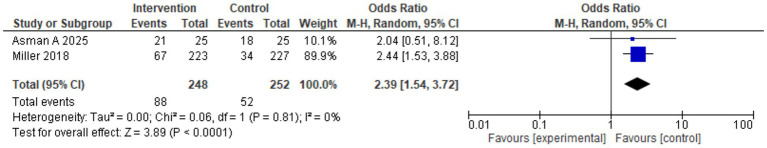
Forest plot for knowledge.

**Figure 9:**
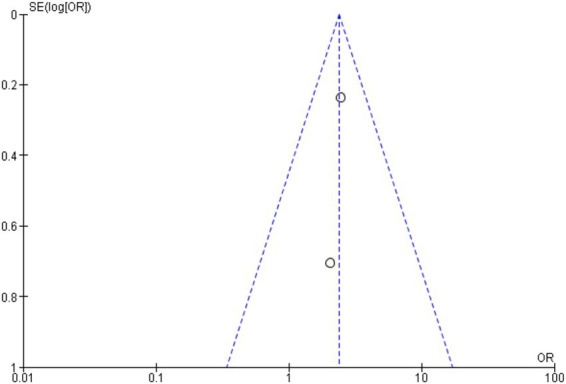
Funnel plot for knowledge.

With only two included studies, heterogeneity and publication-bias assessments are not statistically reliable. The absence of observed heterogeneity should not be interpreted as proof of consistency, and the pooled estimate should be considered preliminary (Figures 8, 9).

##### 3.6.5.2 Behaviour

A total of 2 studies with 140 participants (70 in the intervention group and 70 in the control group) were included. The pooled effect estimate calculated using a random-effects Mantel–Haenszel model showed an odds ratio (OR) of 3.57 with a 95% confidence interval (CI) of 1.60 to 7.97. The overall effect was statistically significant (Z = 3.11, *p* = 0.002).

**Figure 10:**
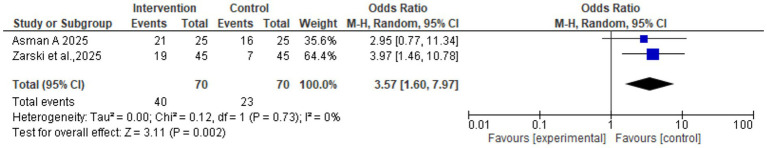
Forest plot for behaviour.

**Figure 11:**
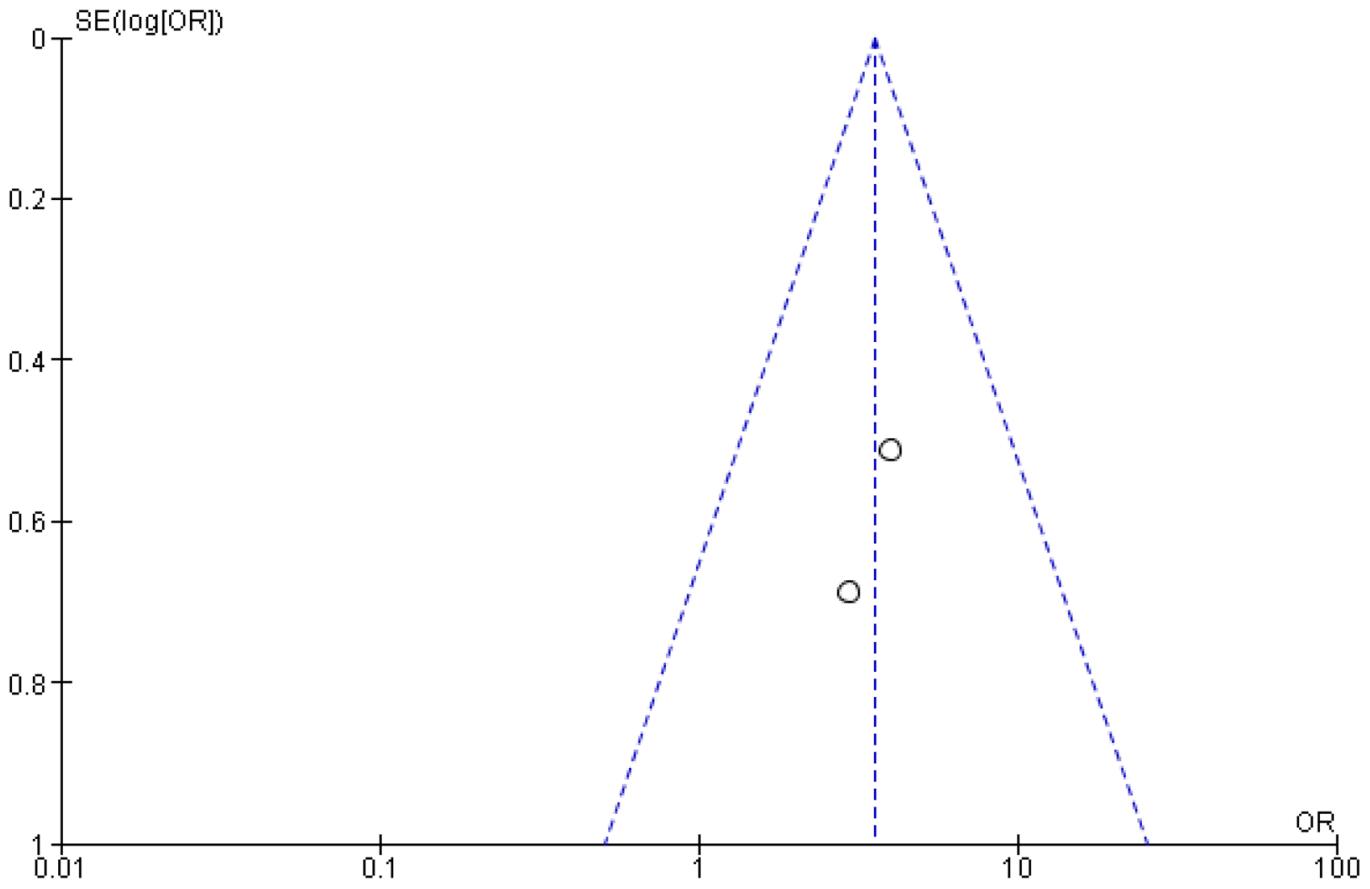
Funnel plot for behaviour.

With only two included studies, heterogeneity and publication-bias assessments are not statistically reliable. The absence of observed heterogeneity should not be interpreted as proof of consistency, and the pooled estimate should be considered preliminary (Figures 10, 11; Tables 6–8).

**Table 6:** Prediction interval.

Outcome	Type	Pooled (%)	95% CI	I^2^ (%)	95% prediction interval
Knowledge	Continuous	0.25	−0.04 to 0.54	92	−0.83 to 1.29
Behaviour	Continuous	–	–	–	–
Knowledge	Dichotomous	–	–	–	–
Behaviour	Dichotomous	3.57	1.60 to 7.97	0	Not estimable*

*Indicates the number of included studies was insufficient to calculate a meaningful prediction interval.

**Table 7:** Leave-one-out sensitivity analysis for knowledge outcome.

Study removed	Pooled estimate (%)	95% CI	I^2^ (%)	Interpretation
None (original)	0.25	−0.04 to 0.54	92	Baseline model with very high heterogeneity.
Areemit et al. (2023) (32)	0.25	−0.08 to 0.57	92	No meaningful change in pooled estimate or heterogeneity; study not influential.
Balbaa et al. (2026) (53)	0.33	0.05 to 0.62	91	Slight increase in pooled effect and CI becomes statistically significant; minor influence but heterogeneity remains high.
Cunningham et al. (2024) (33)	0.22	−0.11 to 0.56	93	Minimal change in pooled estimate; heterogeneity unchanged; not influential.
Holst et al. (2022) (44)	0.19	−0.16 to 0.55	93	Small reduction in pooled effect; heterogeneity unchanged; not influential.
Lam and Lam (2024) (37)	0.22	−0.13 to 0.56	93	No substantial impact on pooled effect or heterogeneity; not influential.
Li et al. (2024) (50)	0.14	−0.16 to 0.44	92	Moderate reduction in pooled estimate but remains within overlapping CI; not influential.
Muller et al. (2017) (35)	0.15	−0.13 to 0.43	89	Slight reduction in heterogeneity (92% → 89%); minor influence on variability but pooled effect stable.
Ngiam et al. (2022) (43)	0.17	−0.15 to 0.48	93	Minimal change; heterogeneity unchanged; not influential.
Singh et al. (2025) (52)	0.34	0.06 to 0.62	92	Moderate increase in pooled effect and CI becomes statistically significant; may exert some influence but heterogeneity persists.
Yang et al. (2025) (49)	0.30	0.00 to 0.60	92	Slight increase in pooled estimate; borderline significance; limited influence overall.

**Table 8:** Leave-one-out sensitivity analysis for behaviour outcome.

Study removed	Pooled estimate (%)	95% CI	I^2^ (%)	Interpretation
None (original)	3.57	1.60 to 7.97	0	Baseline model with no observed heterogeneity.
Asman et al. (2025) (45)	3.97	1.46 to 10.78	-	Minimal change in pooled effect; heterogeneity remains absent; not influential.
Zarski et al. (2025) (55)	2.95	0.77 to 11.34	-	Slight reduction in pooled estimate with wider CI; overall interpretation unchanged; not influential.

#### 3.6.6 Publication bias

##### 3.6.6.1 Funnel plot inspection

Visual inspection of funnel plots suggested approximate symmetry for knowledge/literacy outcomes, with no obvious clustering of smaller studies on one side. However, interpretation remains cautious because the number of studies was small. For behavioural outcomes (k = 2), funnel-plot patterns are not informative for assessing publication bias.

##### 3.6.6.2 Egger’s and Begg’s tests

Formal tests for small-study effects are underpowered when k < 10. Accordingly, Egger’s and Begg’s tests were interpreted only for the knowledge/literacy analysis (k = 10), where no clear evidence of asymmetry was detected. These tests were not performed for behavioural outcomes because the number of studies was too small.

##### 3.6.6.3 Trim-and-fill analysis

**Figure 12:**
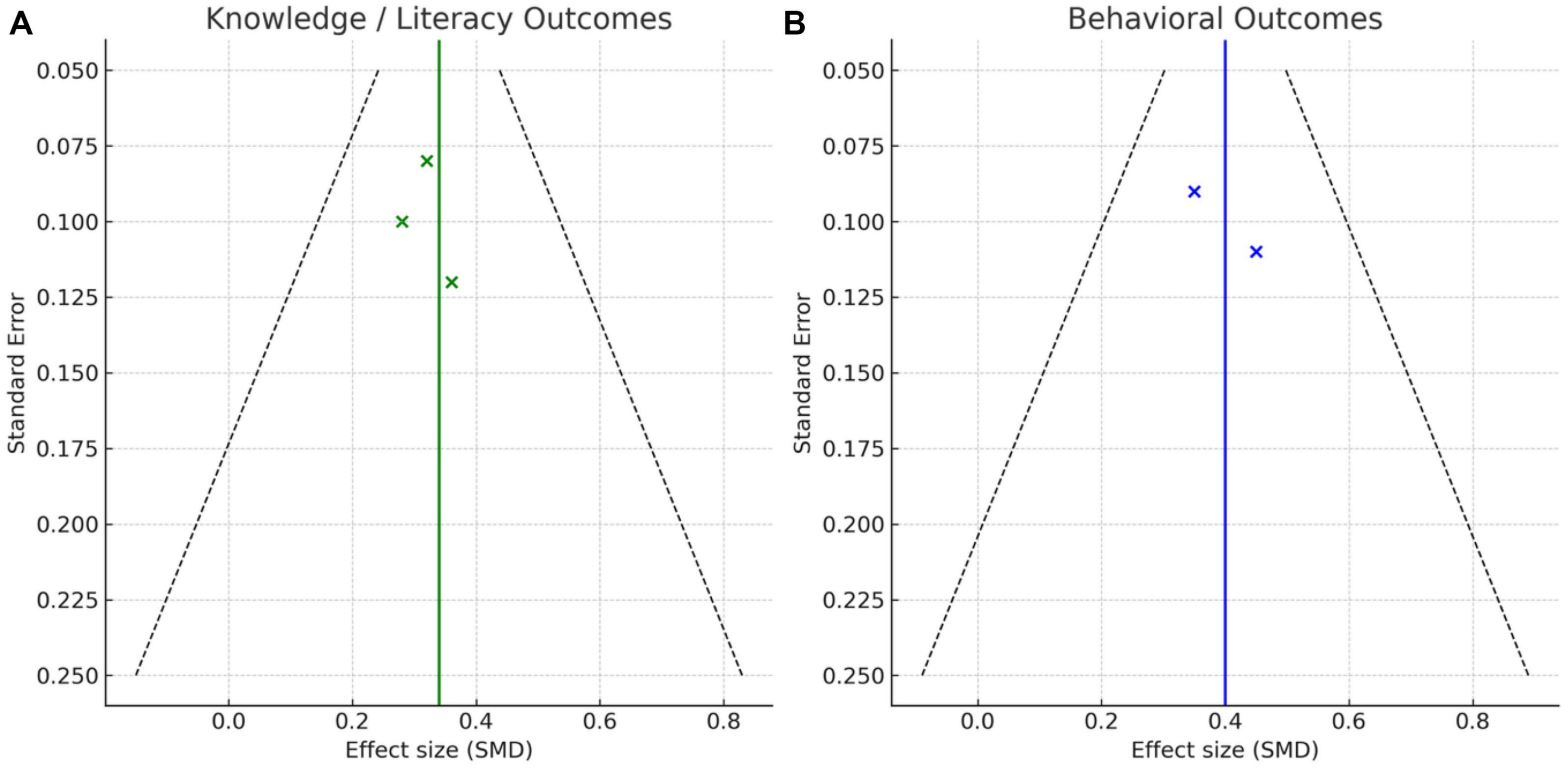
Funnel plots for assessment of publication bias. Funnel plots of individual study effect sizes against their standard errors for **(A)** knowledge/literacy outcomes and **(B)** behavioural outcomes. The vertical solid line represents the pooled effect size, and dashed diagonal lines indicate pseudo-95% confidence limits. The plots did not show obvious asymmetry, but the small number of studies means that funnel-plot inspection, formal tests, and trim-and-fill analyses should be regarded as exploratory. Absence of clear asymmetry should not be interpreted as absence of publication bias.

Exploratory trim-and-fill analysis for knowledge/literacy outcomes did not impute additional studies, and the pooled effect size was unchanged. Nevertheless, publication bias cannot be excluded, particularly because grey literature and unpublished studies were not systematically searched (see Table 9; Figure 12).

**Table 9:** Publication bias assessment.

Outcome domain	No. of studies (k)	Egger’s test (β, p)	Begg’s test (τ, p)	Trim-and-fill adjustment	Adjusted pooled effect (95% CI)	Visual inspection	Interpretation
Knowledge	10	β = −3.328, *p* = 0.25	τ = −0.378, *p* = 0.13	None	SMD = 0.25 (−0.04–0.54)	Symmetric scatter around pooled SMD	Approximate symmetry, but k ≤ 10 → tests underpowered
Behaviour	2	Not performed (k < 10)	Not performed (k < 10)	Not performed	Not applicable	Not interpretable	Too few studies to assess small-study effects reliably.

Egger’s test evaluates funnel plot asymmetry; Begg’s test is based on rank correlation (Kendall’s τ). Both are underpowered when k < 10. Trim-and-fill did not add imputed studies, leaving pooled estimates unchanged. For Publication bias the minimum number of studies > = 10 studies.

### 3.7 Certainty of evidence (GRADE)

**Figure 13:**
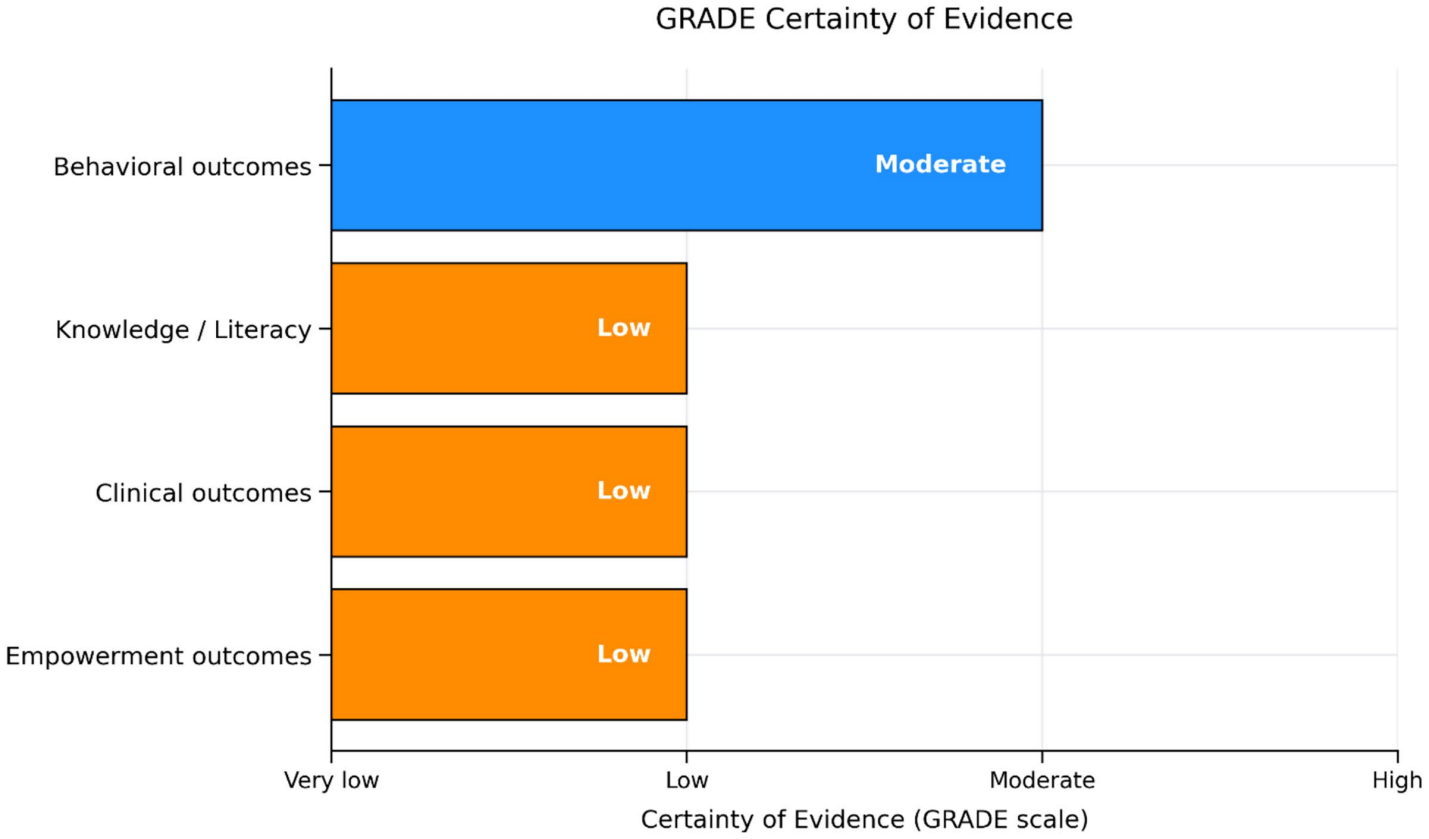
Certainty of evidence across outcome domains (GRADE).

The certainty of evidence, assessed using the GRADE approach, ranged from low to moderate across outcome domains. Evidence for knowledge/literacy outcomes was rated low certainty because of substantial heterogeneity (I^2^ = 92%), imprecision with confidence intervals crossing the null, variation in outcome instruments, and risk of bias in several studies. Behavioural outcomes demonstrated moderate certainty only for selected dichotomous outcomes (OR = 3.57, 95% CI 1.60 to 7.97), but this rating should be interpreted in light of the very small number of contributing studies. Evidence for clinical and empowerment outcomes was rated low certainty, as these domains were informed by fewer than three studies each, heterogeneous measures, and small sample sizes. Overall, certainty was limited by methodological variability, sparse data for several modalities, and publication or language bias that could not be confidently excluded (see Table 10; Figure 13).

**Table 10:** GRADE summary of findings.

Outcome domain	No. of studies/participants	Effect (SMD/OR, 95% CI)	Direction of effect	Certainty (GRADE)	Reasons for downgrading/comments
Knowledge/Literacy	10 studies/1,348 participants	SMD 0.25 (−0.04 to 0.54)	Small, non-significant improvement	Low	Downgraded for: very high heterogeneity (I^2^ = 92%), imprecision (CI crosses 0), risk of bias (subjective outcomes, lack of blinding). No upgrade criteria met.
Behaviour (CRC screening, PA)	2 studies/248 participants	OR 3.57 (1.60 to 7.97)	Clear improvement in behaviour	Moderate	Small k; indirectness; consistency estimates unstable with k = 2.
Clinical outcomes (QoL, distress, GW)	<3 studies/~500 participants	Not pooled	Mixed effects with small single-study evidence	Low	Sparse data, heterogeneity in clinical measures, imprecision, indirectness.
Empowerment outcomes	<3 studies/~400 participants	Not pooled	Inconsistent effects	Low	Sparse evidence, indirectness, inconsistent measures, small sample sizes.
Overall evidence across all domains	12 studies pooled	—	Consistent positive direction	Not graded	Interpret overall certainty domain-by-domain; limited by heterogeneity and sparse modality-specific data.

CI – Confidence Interval; SMD – Standardized Mean Difference; OR – Odds Ratio; I^2^ – Inconsistency statistic (heterogeneity); τ^2^ – Between-study variance; HL – Health Literacy; DHL – Digital Health Literacy; PA – Physical Activity; QoL – Quality of Life.

This figure presents the GRADE certainty ratings across four outcome domains. Knowledge/literacy, clinical, and empowerment outcomes were graded as low certainty, reflecting heterogeneity, small sample sizes, imprecision, and methodological limitations. Behavioural outcomes were graded as moderate certainty only for selected outcomes and should still be interpreted cautiously because few studies contributed data.

## 4 Discussion

### 4.1 Summary of findings

This systematic review and meta-analysis synthesised evidence from 30 interventional studies evaluating digital health communication tools including mobile applications, web-based platforms, social media interventions, telehealth/video tools, and emerging AI-enabled solutions across diverse populations and settings. Included populations spanned multiple regions and health conditions, including preventive screening, chronic disease self-management, reproductive and maternal health, mental health, cancer, cardiovascular care, and oral health. Overall, the findings suggest that digital communication interventions may improve health knowledge and support selected behaviour changes, but the strength of inference is limited by heterogeneity, small pooled samples for several outcomes, and methodological limitations.

Across 10 studies reporting continuous knowledge or literacy outcomes, pooled analysis indicated a small, non-significant overall effect, with high heterogeneity reflecting variability in measurement tools, populations, follow-up duration, and intervention formats. Individual studies targeting parental literacy, menstrual health literacy, and workplace mental health literacy showed directionally positive improvements, but these findings should not be generalized across all digital communication tools. Behavioural outcomes showed statistically significant effects in dichotomous analyses, but these estimates were based on very few studies; therefore, they should be interpreted as promising but preliminary evidence rather than definitive proof of effectiveness.

Narrative synthesis further indicates that mobile apps and structured web-based programs provide the most consistent benefits, especially in preventive care, chronic disease management, and women’s health. AI-enabled interventions appear promising, but the available evidence is sparse and includes protocol/design work as well as small pilot evaluations, which prevents robust inference about AI-specific effectiveness. Social media and telehealth/video interventions were also underrepresented, so their apparent benefits should be interpreted as preliminary rather than definitive. Given the limited number of contributing studies and overlapping confidence intervals, these patterns cannot be considered evidence of differential effectiveness.

### 4.2 Clinical implications

The results have important implications for clinical practice and health systems, but implementation should proceed with caution. Integration of digital health communication tools, particularly mobile apps and web platforms, into preventive care pathways may facilitate screening uptake and healthier behaviours such as physical activity (59, 60). These tools may also support scalable patient education and engagement in resource-poor settings (61–63). AI-enabled tools may provide opportunities for personalized health messaging and adaptive decision support (64–66), but this review identified too little direct evidence to draw firm conclusions about their added effect relative to conventional digital tools (67). Similar caution applies to telehealth/video and social media modalities, which were underrepresented. Although findings across underserved and low socioeconomic status populations suggest potential to reduce disparities (68), digital interventions could also widen inequities if access, usability, language, and digital literacy barriers are not addressed. These tools should therefore be regarded as adjuncts for education and engagement rather than alternatives to clinical care, pending more robust evidence of sustained clinical benefit (69, 70).

### 4.3 Strength and limitations

Strengths of this review include a comprehensive search strategy across multiple databases, registration and reporting in line with PRISMA and PROSPERO, duplicate screening and risk-of-bias assessment, and quantitative synthesis using random-effects models. Cochrane methods for risk of bias assessment and GRADE evaluation were applied, strengthening methodological rigor. Several limitations should be noted. First, heterogeneity was substantial and arose from differences in intervention modality, intensity, comparator type, target population, follow-up duration, and outcome measurement tools; therefore, pooled estimates should be interpreted as broad summary signals rather than precise estimates of a uniform intervention effect. Second, several pooled analyses included only a small number of studies, especially for behavioural outcomes and subgroup analyses, limiting power and making heterogeneity, publication-bias testing, and comparative subgroup interpretation unreliable. Third, some interventions contained overlapping features, such as AI embedded within mobile or web-based delivery, which limits attribution of effects to any single component even though a dominant-modality rule was used. Fourth, social media, telehealth/video, and AI-enabled modalities were underrepresented, so findings for these approaches remain preliminary. Fifth, several included studies had moderate-to-high risk of bias, lack of participant blinding was unavoidable, and self-reported outcomes were vulnerable to performance, detection, and social-desirability bias. Finally, the review was restricted to English-language, peer-reviewed publications, and grey literature, conference abstracts, dissertations, preprints, and unpublished studies were not systematically searched; publication and language bias therefore cannot be excluded. Additionally, DHL was rarely measured directly, so conclusions about true digital health literacy remain limited.

### 4.4 Future directions

Future research should prioritize standardized measurement of digital health literacy using validated tools, such as eHEALS (71) or the Digital Health Literacy Instrument (DHLI) (72), and should clearly distinguish digital literacy, disease-specific knowledge, behaviour change, empowerment, and clinical endpoints. Larger, adequately powered randomized trials are needed to evaluate AI-enabled interventions, with explicit reporting of what AI component was used, how it influenced personalization or decision support, and whether it adds benefit beyond conventional digital tools. Targeted evaluations are also needed for underrepresented modalities such as social media and telehealth/video delivery. Future studies should predefine core outcomes, use objective behavioural and clinical measures where possible, include longer follow-up, and report implementation context to improve comparability. Equity-focused design should address barriers among populations with low literacy or limited digital access through usability testing, language adaptation, accessibility standards, and culturally tailored content. Implementation-science approaches are also needed to examine integration into routine care, including provider training, workflow adaptation, governance, privacy, and cost-effectiveness.

## 5 Conclusion

This systematic review and meta-analysis suggests that digital health communication interventions may improve patient knowledge and selected behavioural outcomes, but the evidence remains cautious rather than definitive. Continuous knowledge/literacy outcomes showed a small, statistically non-significant effect and substantial heterogeneity, while significant behavioural findings were based on only a small number of studies. Evidence for AI-enabled, telehealth/video, and social-media interventions is especially limited, and clinical and empowerment outcomes remain too heterogeneous for firm conclusions. Current evidence supports the potential role of digital health communication tools as adjuncts for patient education and engagement, not as established stand-alone solutions. Further large-scale, methodologically robust trials using standardized outcome measures and direct digital health literacy assessment are required before firm conclusions about effectiveness can be drawn.

## Data Availability

The original contributions presented in the study are included in the article/Supplementary material, further inquiries can be directed to the corresponding author.
