# Correction: Enhancing patient knowledge and behaviour through digital health communication: a systematic review and random-effects meta-analysis of mobile, web-based, social media, telehealth, and AI-enabled interventions

**DOI:** 10.3389/fpubh.2026.1921630

**Published:** 2026-07-24

**Authors:** Asma Alshanqiti, Hadel Alghabban, Amal Mohammed Q. Surrati, Sara Ibrahim, Wejdan Owaydhah

**Affiliations:** 1 Department of Family and Community Medicine and Medical Education, Taibah University College of Medicine, Medina, Saudi Arabia; 2 Department of Basic Medical Sciences, Taibah University College of Medicine, Medina, Saudi Arabia

**Keywords:** artificial intelligence, digital health communication, digital health literacy, meta-analysis, mobile apps, patient empowerment, social media, systematic review

Affiliation “Department of Basic Medical Sciences, Taibah University College of Medicine, Medina, Saudi Arabia” was omitted from the paper. This affiliation has now been added for authors Hadel Alghaban, Sara Ibrahim, and Wejdan Owaydhah, replacing affiliation 1.

The original version of this article has been updated.

