# Supplementary material for: Enhancing patient knowledge and behaviour through digital health communication: a systematic review and random-effects meta-analysis of mobile, web-based, social media, telehealth, and AI-enabled interventions
Source: Front Public Health. 2026 Jun 24;14:1741936. doi: 10.3389/fpubh.2026.1741936 (PMC13342081; doi:10.3389/fpubh.2026.1741936)
Supplement: Supplementary file 1 [file Table_1.DOCX]

**Retained references with reasons:**

| **No.** | **Author,year** | **Reference** | **Reason** |
| --- | --- | --- | --- |
| 1 | Adam M. et al. (2023) | Adam M, Kwinda Z, Dronavalli M, Leonard E, Nguyễn VK, Tshivhase V, et al. Effect of Short, Animated Video Storytelling on Maternal Knowledge and Satisfaction in the Perinatal Period in South Africa: Randomized Controlled Trial. J Med Internet Res [Internet]. 2023 Oct 13;25:e47266. Available from: https://www.jmir.org/2023/1/e47266 | Directly aligns with digital communication improving health knowledge. |
| 2 | Ambrosi E. et al. (2025) | Ambrosi E, Mezzalira E, Canzan F, Leardini C, Vita G, Marini G, et al. Effectiveness of digital health interventions for chronic conditions management in European primary care settings: Systematic review and meta-analysis. Int J Med Inform [Internet]. 2025 Apr;196:105820. Available from: https://linkinghub.elsevier.com/retrieve/pii/S1386505625000371 | Meta-analysis on digital health effectiveness in primary care. |
| 3 | Chaturvedi U. et al. (2025) | Chaturvedi U, Chauhan SB, Singh I. The impact of artificial intelligence on remote healthcare: Enhancing patient engagement, connectivity, and overcoming challenges. Intelligent Pharmacy [Internet]. 2025 Oct;3(5):323–9. Available from: https://linkinghub.elsevier.com/retrieve/pii/S2949866X24001230 | Links AI to patient engagement and remote care. |
| 4 | Chioma Anthonia et al. (2024 | Chioma Anthonia Okolo, Oloruntoba Babawarun, Tolulope Oyinlola Olorunsogo. MOBILE HEALTH (MHEALTH) INNOVATIONS FOR PUBLIC HEALTH FEEDBACK: A GLOBAL PERSPECTIVE. International Medical Science Research Journal [Internet]. 2024 Mar 17;4(3):235–46. Available from: https://fepbl.com/index.php/imsrj/article/view/915 | Offers global perspective on mHealth for public health feedback. |
| 5 | Cho H. et al. (2018) | Cho H, Yu B, Cannon J, Zhu YM. Efficacy of a Media Literacy Intervention for Indoor Tanning Prevention. J Health Commun [Internet]. 2018 Jul 3;23(7):643–51. Available from: https://www.tandfonline.com/doi/full/10.1080/10810730.2018.1500659 | Behavioral intervention using media literacy; supports communication-based behavior change. |
| 6 | Choi J. et al. (2023) | Choi J, Choi S, Song K, Baek J, Kim H, Choi M, et al. Everyday Digital Literacy Questionnaire for Older Adults: Instrument Development and Validation Study. J Med Internet Res [Internet]. 2023 Dec 14;25:e51616. Available from: https://www.jmir.org/2023/1/e51616 | Instrument development relevant to measuring literacy. |
| 7 | de Figueirêdo RC. et al. (2024) | de Figueirêdo RC, de Siqueira Silva Í, de Araújo AJ, Silva CRDV, Martiniano CS, Brito EWG, et al. Preparation and validation of the instrument “QualiAPS digital—Brazil” for assessing digital health care in primary health care: a required tool. Front Public Health [Internet]. 2024 Jul 16;12. Available from: https://www.frontiersin.org/articles/10.3389/fpubh.2024.1304148/full | Supports measurement and evaluation of digital health literacy. |
| 8 | Fahim YA. et al. (2025) | Fahim YA, Hasani IW, Kabba S, Ragab WM. Artificial intelligence in healthcare and medicine: clinical applications, therapeutic advances, and future perspectives. Eur J Med Res [Internet]. 2025 Sep 23;30(1):848. Available from: https://eurjmedres.biomedcentral.com/articles/10.1186/s40001-025-03196-w | Provides clinical context for AI applications in health. |
| 9 | Fitzpatrick PJ (2023) | Fitzpatrick PJ. Improving health literacy using the power of digital communications to achieve better health outcomes for patients and practitioners. Front Digit Health [Internet]. 2023 Nov 17;5. Available from: https://www.frontiersin.org/articles/10.3389/fdgth.2023.1264780/full | Supports digital communication’s role in improving health literacy. |
| 10 | Glatz T. et al. (2023) | Glatz T, Tops W, Borleffs E, Richardson U, Maurits N, Desoete A, et al. Dynamic assessment of the effectiveness of digital game-based literacy training in beginning readers: a cluster randomised controlled trial. PeerJ [Internet]. 2023 Jul 31;11:e15499. Available from: https://peerj.com/articles/15499 | Focuses on reading literacy in children, not health or DHL. |
| 11 | Kim H. et al. (2024) | Kim H, Schnall R, Yoon N, Koh SJ, Lee J, Cheon JH. Development and Validation of a Mobile-Centered Digital Health Readiness Scale (mDiHERS): Health Literacy and Equity Scale. J Med Internet Res [Internet]. 2024 Aug 13;26:e58497. Available from: https://www.jmir.org/2024/1/e58497 | Measures digital health readiness and literacy. |
| 12 | Le C. et al. (2025) | Le C, Søberg Finbråten H, Griebler R, Levin-Zamir D, Guttersrud Ø. Ability to Utilize Digital Health Services: Validation of the Digital HealthCare Scale in Adolescents and Young Adults. HLRP: Health Literacy Research and Practice [Internet]. 2025 Jan;9(1). Available from: https://journals.healio.com/doi/10.3928/24748307-20241204-01 | Validates ability to use digital health services. |
| 13 | Lepore SJ. et al. (2019) | Lepore SJ, Rincon MA, Buzaglo JS, Golant M, Lieberman MA, Bauerle Bass S, et al. Digital literacy linked to engagement and psychological benefits among breast cancer survivors in Internet‐based peer support groups. Eur J Cancer Care (Engl) [Internet]. 2019 Jul 18;28(4). Available from: https://onlinelibrary.wiley.com/doi/10.1111/ecc.13134 | Shows psychological benefits of digital engagement. |
| 14 | Li Q. et al. (2024) | Li Q, Piaseu N, Phumonsakul S, Thadakant S. Effects of a Comprehensive Dietary Intervention Program, Promoting Nutrition Literacy, Eating Behavior, Dietary Quality, and Gestational Weight Gain in Chinese Urban Women with Normal Body Mass Index during Pregnancy. Nutrients [Internet]. 2024 Jan 10;16(2):217. Available from: https://www.mdpi.com/2072-6643/16/2/217 | Digital intervention improving nutrition literacy and outcomes. |
| 15 | Malikhao P. (2020) | Malikhao P. Health Communication: Approaches, Strategies, and Ways to Sustainability on Health or Health for All. In: Handbook of Communication for Development and Social Change [Internet]. Singapore: Springer Singapore; 2020. p. 1015–37. Available from: http://link.springer.com/10.1007/978-981-15-2014-3_137 | Provides theoretical background on health communication. |
| 16 | Malloy JA. et al. (2024) | Malloy JA, Partridge SR, Kemper JA, Braakhuis A, Roy R. Feasibility and Preliminary Efficacy of Co-Designed and Co-Created Healthy Lifestyle Social Media Intervention Programme the Daily Health Coach for Young Women: A Pilot Randomised Controlled Trial. Nutrients [Internet]. 2024 Dec 18;16(24):4364. Available from: https://www.mdpi.com/2072-6643/16/24/4364 | Social media intervention targeting health behavior. |
| 17 | Manganello JA. et al. (2024) | Manganello JA, Colvin KF, Hadley M, O’Brien K. Get Health‘e’: A Pilot Test of a Digital Health Literacy Intervention for Young Adults. HLRP: Health Literacy Research and Practice [Internet]. 2024 Oct;8(4). Available from: https://journals.healio.com/doi/10.3928/24748307-20240723-01 | Pilot study on improving digital health literacy. |
| 18 | Mathews D. et al. (2023) | Mathews D, Abernethy A, Butte AJ, Ginsburg P, Kocher B, Novelli C, et al. Telehealth and Mobile Health: Case Study for Understanding and Anticipating Emerging Science and Technology. NAM Perspectives [Internet]. 2023 Nov 15;11(15). Available from: https://nam.edu/telehealth-and-mobile-health-case-study-for-understanding-and-anticipating-emerging-science-and-technology/ | Provides context for emerging digital health technologies. |
| 19 | Matos Fialho PM. et al. (2025) | Matos Fialho PM, Wenig V, Heumann E, Müller M, Stock C, Pischke CR. Digital public health interventions for the promotion of mental well-being and health behaviors among university students: a rapid review. BMC Public Health [Internet]. 2025 Jul 18;25(1):2500. Available from: https://bmcpublichealth.biomedcentral.com/articles/10.1186/s12889-025-23669-1 | Supports behavioral and mental health outcomes in youth. |
| 20 | Merino M. et al. (2024) | Merino M, del Barrio J, Nuño R, Errea M. Value-based digital health: A systematic literature review of the value elements of digital health care. Digit Health [Internet]. 2024 Jan 13;10. Available from: https://journals.sagepub.com/doi/10.1177/20552076241277438 | Discusses value elements in digital health systems, aligning with your discussion. |
| 21 | Petrič G., Atanasova S. (2024) | Petrič G, Atanasova S. Validation of the extended e-health literacy scale: structural validity, construct validity and measurement invariance. BMC Public Health [Internet]. 2024 Jul 25;24(1):1991. Available from: https://bmcpublichealth.biomedcentral.com/articles/10.1186/s12889-024-19431-8 | Instrument validation for eHealth literacy |
| 22 | Theodosiou AA., Read RC (2023) | Theodosiou AA, Read RC. Artificial intelligence, machine learning and deep learning: Potential resources for the infection clinician. Journal of Infection [Internet]. 2023 Oct;87(4):287–94. Available from: https://linkinghub.elsevier.com/retrieve/pii/S0163445323003791 | Historical and clinical context for AI in healthcare. |
| 23 | Wamala Andersson S. et al. (2025) | Wamala Andersson S, Gonzalez MP. Digital health literacy—a key factor in realizing the value of digital transformation in healthcare. Front Digit Health [Internet]. 2025 Jun 5;7. Available from: https://www.frontiersin.org/articles/10.3389/fdgth.2025.1461342/full | Highlights DHL’s role in realizing digital health value. |

**Newly Added References:**

1. Barony Sanchez RH, Bergeron-Drolet LA, Sasseville M, Gagnon MP. Engaging patients and citizens in digital health technology development through the virtual space. Front Med Technol [Internet]. 2022 Nov 25;4. Available from: https://www.frontiersin.org/articles/10.3389/fmedt.2022.958571/full
2. Borges do Nascimento IJ, Abdulazeem H, Vasanthan LT, Martinez EZ, Zucoloto ML, Østengaard L, et al. Barriers and facilitators to utilizing digital health technologies by healthcare professionals. NPJ Digit Med [Internet]. 2023 Sep 18;6(1):161. Available from: https://www.nature.com/articles/s41746-023-00899-4
3. Chioma Anthonia Okolo, Oloruntoba Babawarun, Jeremiah Olawumi Arowoogun, Adekunle Oyeyemi Adeniyi, Rawlings Chidi. The role of mobile health applications in improving patient engagement and health outcomes: A critical review. International Journal of Science and Research Archive [Internet]. 2024 Feb 28;11(1):2566–74. Available from: https://ijsra.net/content/role-mobile-health-applications-improving-patient-engagement-and-health-outcomes-critical
